# High microbial diversity in the rhizosphere and soil improves carrot (*Daucus carota* L.) postharvest storability

**DOI:** 10.1093/femsec/fiag102

**Published:** 2026-09-07

**Authors:** Sannakajsa Velmala, Taina Pennanen, Minna Haapalainen, Petteri Karisto, Satu Latvala, Minna Pirhonen, Terhi Suojala-Ahlfors

**Affiliations:** Natural Resources Institute Finland, Latokartanonkaari 9, 00790, Helsinki,Finland; Natural Resources Institute Finland, Latokartanonkaari 9, 00790, Helsinki,Finland; Natural Resources Institute Finland, Latokartanonkaari 9, 00790, Helsinki,Finland; University of Helsinki, Faculty of Agriculture and Forestry, Latokartanonkaari 7, 00790, Helsinki,Finland; Natural Resources Institute Finland, Tietotie 4, 31600, Jokioinen,Finland; Natural Resources Institute Finland, Tietotie 4, 31600, Jokioinen,Finland; University of Helsinki, Faculty of Agriculture and Forestry, Latokartanonkaari 7, 00790, Helsinki,Finland; Natural Resources Institute Finland, Toivonlinnantie 518, 21500, Piikkiö,Finland

**Keywords:** carrot storage loss, soil fungal community, soil bacterial community, *Mycocentrospora acerina*, *Metarhizium* sp

## Abstract

Postharvest diseases can cause significant losses in carrot (*Daucus carota*) yield during storage. In this study, the effects of soil quality and microbial diversity in bulk field soil and the rhizosphere on carrot postharvest storability were investigated. Microbial genera in bulk soil and rhizosphere samples were identified by Illumina MiSeq amplicon sequencing of the bacterial 16S rRNA gene and fungal ITS regions. Disease symptoms in the stored carrots were monitored after 6 months of cold storage, and the main pathogens were identified. Field sites with mineral soils, which had higher pH values, had greater bacterial and fungal diversity than sites with organic soils. Organically farmed fields had greater bacterial diversity than conventionally farmed fields. The community structures of both bacteria and fungi were largely driven by field site, including its main chemical characteristics, with rhizosphere and bulk soil samples from the same site displaying comparable assemblages. Higher soil fungal diversity was associated with greater postharvest storability of carrots. Weather during the growing season also affected the observed fungal biodiversity, which was higher at sites with lower temperatures and less rain prior to harvest.

## Teaser

High microbial diversity, particularly within fungal communities, combined with the predominance of functionally important bacterial taxa, was associated with improved postharvest storability of carrots.

## Introduction

Soil health encompasses the soil’s physical, chemical, and biological condition and determines its capacity to function as a vital living system and to provide ecosystem services (Larkin 2015, Directorate-General for Environment 2023). Healthy soil is defined by its ongoing ability to function as a living system, sustain productivity, protect air and water quality, and support plant, animal, and human health (Pankhurst et al. 1997). The importance of healthy soil in mitigating greenhouse gases and facilitating food production cannot be overstated. Globally, a considerable proportion of agricultural soils are degraded as a result of negative changes in their physical, chemical, and biological condition (Lal 2015), resulting in diminished productivity due to weakened soil ecosystem services (Pereira et al. 2018). This suggests that early signs of declining soil health, even before measurable changes in soil physical properties can be observed, may predict an increased incidence of disease, especially under intensive cropping.

The rhizosphere, defined as the interaction zone between plant roots and soil, including all its microbial diversity, is of critical importance for plants, as it governs their water and nutrient uptake, which is required for growth and development. The rhizosphere is the zone of direct root contact for beneficial microorganisms, including plant growth-promoting bacteria and symbiotic mycorrhizal fungi (Raaijmakers et al. 2009). In addition to beneficial microbes, the rhizosphere microbiome can also contain pathogens (Mendes et al. 2013, Mesny et al. 2023). The structure and functionality of the rhizosphere microbial populations are also shaped by the plant species and genotype and by the soil type (Berg and Smalla 2009). It has been suggested that plants actively recruit beneficial microorganisms to improve their resistance against various stress factors (Mesny et al. 2023). In disease suppressive soils, the antimicrobial compounds produced by rhizosphere microorganisms suppress pathogen populations in the soil (Raaijmakers et al. 2009). Moreover, the beneficial microorganisms can enhance the plant defence system against pathogens. Recent studies have demonstrated that even in soils which are chemically and structurally relatively similar, the composition of soil microbiota can have a significant impact on crop health and yield (Ali et al. 2023).

Plants interact with a plethora of different microorganisms that colonize all the plant surfaces (Mesny et al. 2023). Bacterial species that have previously been linked to the suppression of disease are found in several phyla, including Pseudomonadota (synonym Proteobacteria, new phylum names by Oren et al. 2021), Bacillota (syn. Firmicutes), and Actinomycetota (syn. Actinobacteria) (Mendes et al. 2011, Kinkel et al. 2012). Moreover, both biotic and abiotic environmental factors induce changes in the prevalence of different microbial species that may suppress or promote plant diseases. Vegetable decay during storage is frequently attributable to the interaction of decreased biodiversity and augmented proportions of pathogenic species (Kusstatscher et al. 2020).

Carrot (*Daucus carota* subsp. *sativa*) is a globally important field vegetable, with the seventh-highest production volume (Que et al. 2019). In Northern Europe, where the cultivation of field vegetables is confined to a single annual growing season, most of the carrot yield is typically stored prior to marketing. However, during the storage period, which may last up to 6 months, a significant proportion of the yield is lost, mostly due to deterioration by pathogenic microbes. As demonstrated by research conducted in Finland by Latvala et al. (2024), postharvest storage losses of carrots due to decay can be _~_20%, even under well-controlled storage conditions. An earlier study in Norway by Hermansen et al. (2012) estimated losses of between 20% and 50%, resulting in considerable economic losses.

In the boreal region, soil microbial communities are strongly shaped by management practices, including farming system and crop rotation (Peltoniemi et al. 2021). Many fungal genera, including plant pathogens, such as *Rhizoctonia* and *Fusarium*, are predominant members of the core mycobiome of carrot (Abdelrazek et al. 2020). Both the wounds caused by harvesting, and the water loss during extended postharvest storage render the carrot root more vulnerable to pathogens. In Finland, the predominant pathogenic fungi responsible for postharvest decay of carrots are *Mycocentrospora acerina*, causing liquorice rot, *Botrytis cinerea*, causing grey mould, and *Fusarium* spp., which are often considered secondary pathogens (Suojala 1999, Latvala et al. 2024). Furthermore, different bacterial strains of the genera *Pseudomonas, Pectobacterium*, and *Erwinia* have also been shown to cause decay of carrots during storage (Kahala et al. 2012). In sugar beets (*Beta vulgaris* L.), the core microbiome was found to differ between healthy and diseased plants, with high proportions of antagonistic bacteria providing resistance to deleterious shifts in the fungal microbiome (Kusstatscher et al. 2019). Thus, the plant microbiome was proposed to be the key element in biological control of postharvest pathogens and storability of root vegetables (Kusstatscher et al. 2020).

Despite extensive research on plant-associated microbial communities, their role in carrot health during storage remains unclear. The present study investigates the influence of soil and rhizosphere microbes on carrot storability and postharvest diseases. We hypothesized that (i) both beneficial and potentially pathogenic bacteria and fungi are found in the rhizosphere and bulk soil surrounding carrot roots, (ii) high microbial diversity reduces the occurrence of postharvest diseases, and (iii) good storability is associated with a high abundance of beneficial microbial groups. Samples were collected from 26 carrot fields in Finland. The samples were analysed to assess soil characteristics and bacterial and fungal diversity using sequencing of the bacterial 16S rRNA gene and fungal ITS2 regions. Storage losses of carrots grown in these fields were monitored over 6 months of cold storage. Furthermore, we investigated whether initial indications of declining soil health were predominantly associated with biological rather than physical or chemical characteristics.

## Materials and methods

### Carrot and soil sampling

Carrot and soil samples were taken from mid-September to early October in 2020 and 2021 from 14 and 12 field sites, respectively (Table 1; Supplementary Fig. S1; A–Z). All field sites sampled in 2021 differed from those sampled in 2020. In both years, three field sites were organically farmed according to the European Union’s organic farming rules, which prohibit the use of synthetic pesticides and mineral fertilizers and emphasize long-term soil fertility, biodiversity, and ecosystem health. All organic farms and a few conventional farms used clover ley, white sweet clover (*Melilotus albus*), white mustard (*Sinapis alba*), green manure, and digestate in rotation. Sampling was performed in six replicate areas per field. The distance between separate sampling areas was 30–100 m, depending on field size. Each sampling area comprised 5 m of a planted carrot row containing 200–250 plants. The fields used in this study were commercial carrot production fields reflecting actual soil types and farming practices. This approach ensured that the results represented genuine production conditions, although it did not provide a fully balanced experimental design.

**Table 1:** Site characteristics across 26 carrot fields, including management [farm type Con/Org, carrot in history (A), green manure used (B), variety (Maestro M, Romance R, Newhall N)], soil properties (electrical conductivity (10 mS cm^−1^), cation exchange capacity (CEC) (cmol/kg), nutrient concentrations (mg/l)), weather (mean temperature in the month before sampling; mean rainfall in the week and month before harvest), and microbial diversity (richness and Shannon index) in rhizosphere and bulk soil.

Code	Year	Management	Soil type	OM%	pH	Cond	CEC	Ca	P	K	Mg	S	N	Yield	Storability	Temp.	Precipitation	Bacterial obs R, S	Bacterial Shan R, S	Fungal obs R, S	Fungal Shan R, S
A	2020	Con, M	mull	23.5	5.8	4.3	26	3100	7.3	150	320	150	11.4	105.3	76.7	13.15	wk = 4.74, mo = 78.9	302, 329	3.9, 4.1	2271, 2082	7, 6.9
B		Con, R	clay loam	9.3	6.3	3.2	21	3200	24	140	180	110	12.1	68.8	94.8	13.16	wk = 1.24, mo = 64.3	252, 271	4.1, 4.2	1994, 1849	6.8, 6.6
C		Con A, R	mull	22.4	5.6	3.4	19	2100	9	98	220	120	22.6	104.5	84.6	14.56	wk = 2.52, mo = 58.7	289, 298	4, 4	1944, 1806	6.7, 6.6
D		Org A, B, M	sandy medium fine sand	4.1	6.8	0.9	12	1800	53	100	160	10	19.1	69.3	62	11.09	wk = 0.3, mo = 83.6	280, 243	3.8, 3.6	2645, 2595	7.2, 7.1
E		Org A, B, M	carex peat	53.6	5.9	1.6	22	2800	5.9	170	220	23	20.5	40.5	35	10.85	wk = 1.48, mo = 81.1	294, 289	4, 4	2427, 2309	7.1, 7
F		Con A, B, M	medium fine sand	4.1	6.4	0.9	8	1100	15	120	120	25	<10	61	66.1	10.88	wk = 0.9, mo = 84.9	361, 306	4.4, 4.3	2485, 2527	7.1, 7.1
G		Con A, B, R	medium fine sand	5.2	6.7	0.8	13	1900	43	170	160	11	10	78.5	90.4	11.18	wk = 0.27, mo = 83.6	321, 308	4.3, 4.3	2604, 2569	7.1, 7.1
H		Con A, R	silty clay	15.9	6.1	1.9	18	2600	5.4	95	180	35	21.9	78.5	79.2	12.17	wk= 0.91, mo = 18.3	298, 342	4.1, 4.4	2275, 2129	7, 6.9
I		Con A, R	mull	37.6	5.6	3.1	38	4200	5.2	180	460	67	31	75.5	68.7	12.23	wk = 0.47, mo = 44.3	242, 326	3.5, 4.1	1926, 1838	6.6, 6.6
J		Con A, R	mull	38.5	5.8	2.5	37	4500	4	120	440	35	32.4	83.9	69.7	12	wk = 1.12, mo = 23.4	276, 315	3.9, 4.1	1923, 1823	6.7, 6.6
K		Org A, B, R	fine sandy till	4.5	6.6	0.7	11	1600	14	140	100	11	<10	28.7	66.3	12.27	wk = 0.08, mo = 44.5	331, 355	4.3, 4.3	2527, 2416	7.2, 7.2
L		Con, M	carex peat	80	5.4	7	46	5000	10	64	300	190	40.2	83	92.6	13.02	wk = 3.25, mo = 66.1	309, 337	4, 3.9	2120, 1972	6.9, 6.7
M		Con, M	mull	32.9	5.3	2.8	26	2500	8.3	120	220	130	10	85.2	48.3	13.17	wk = 4.54, mo = 76.9	203, 246	3.4, 3.7	1996, 1831	6.8, 6.6
N		Con, M	mull	29.8	5.1	3.4	27	2300	4.5	74	190	74	43	96.5	87.9	12.88	wk = 1.08, mo = 53.6	333, 335	4.1, 4.2	1867, 1695	6.7, 6.5
O	2021	Con A, B, R	medium fine sand	6.1	5.6	2	11	1200	13	190	100	43	42.5	47.5	91.1	9.3	wk = 0.05, mo = 29.4	386, 350	4.6, 4.5	2395, 2377	7, 7
P		Org A, B, M	fine sandy till	8.9	6.5	0.8	19	2700	18	210	270	13	<10	48.7	46.5	7.78	wk = 0.95, mo = 33.4	362, 339	4.3, 4.2	2753, 2638	7.2, 7.2
Q		Con A, M	mull	27.1	6.5	3.5	33	4800	11	150	510	110	14	74.2	86.7	11.24	wk = 0, mo = 19.1	364, 344	4.4, 4.4	2209, 2277	6.9, 7
R		Con, N	clay loam	7.2	6.6	2.2	22	3300	72	170	280	67	<10	92.1	99.5	10.23	wk = 4.1, mo = 46.6	292, 319	3.8, 4.1	1967, 1891	6.8, 6.8
S		Con A, M	mull	22.7	5.2	4	25	2300	7.9	170	180	150	30.1	46	69.4	10.06	wk = 2.81, mo = 60.3	317, 355	4.1, 4.3	1523, 1584	6.3, 6.4
T		Con A, M	loam	10.3	7	3	24	4000	98	270	270	99	14	59.3	98.4	10	wk = 3.7, mo = 42.4	350, 348	4.2, 4.4	2148, 2044	7, 6.9
U		Con, R	mull	34.4	6.2	2.1	40	5500	8	210	550	21	13.3	64.3	86.2	8.82	wk = 1.01, mo = 32	331, 362	4.1, 4.2	2032, 2019	6.8, 6.8
V		ConA, R	mull	33.3	5.7	2.2	32	3800	3.5	180	300	35	25.9	74.9	84.2	8.86	wk = 0.37, mo = 30.9	331, 336	4.2, 4.1	2067, 1991	6.8, 6.7
W		Con, R	carex peat	52.7	5.8	2.3	28	3600	3.6	77	260	35	24.5	79.9	82.2	8.86	wk = 0.37, mo = 30.9	345, 352	4.1, 4.2	1967, 1821	6.7, 6.6
X		Con A, M	carex peat	62.9	5.8	3.3	38	4600	13	74	500	110	14.7	81.1	68.8	10.93	wk = 0, mo = 22.2	408, 409	4.6, 4.6	2224, 2184	6.9, 6.9
Y		Org B, M	fine sandy till	4.8	6.4	0.7	14	2000	13	120	180	14	<10	49.6	92.4	8.78	wk = 0.9, mo = 34.5	476, 408	4.6, 4.3	2711, 2853	7.3, 7.3
Z		Org A, B, M	fine sandy till	3.6	6.7	0.7	10	1500	12	230	130	6.5	<10	39.9	93.7	9.07	wk = 0.85, mo = 35.1	429, 362	4.8, 4.6	2730, 2768	7.2, 7.3

Soil samples for DNA analysis of rhizosphere and bulk soil were collected first. Rhizosphere soil was sampled from 10 sampling points per area by lifting two carrots 50 cm apart. Rhizosphere soil was gently brushed from these 20 carrots into a zip-lock bag using a clean disposable toothbrush. The same toothbrush was used for all carrots within the same sampling area, and the soil detached from the carrots and toothbrush was frozen at −20°C within 24 h. Altogether, six replicate rhizosphere samples were taken per field. In total, 156 rhizosphere samples were collected.

Bulk soil was sampled by collecting six replicate soil cores (1 m apart; diameter 25 mm; depth 0–10 cm) from each sampling area into clean zip-lock bags. The samples were carefully mixed and divided into two subsamples: one for soil property analysis and the other for DNA analysis. The latter sample set was frozen at −20°C within 24 h. Altogether, six replicate bulk soil samples were taken per field. The soil borer was washed with soap and tap water between fields and wiped clean with ethanol between replicate sampling sites.

Additional soil samples (depth 0–25 cm, 400 ml) were taken for soil nutrient analysis. Soil analysis was performed on a composite sample, which was formed by mixing the six subsamples from all six sampling areas of the same field.

After soil sampling, all the carrots from the same sampling area were collected to a plastic container, and the carrot tops were removed. In total, six replicate 8-kg carrot samples, comprising on average 235 carrots, were taken per field and packed into woven polypropylene bags, separately for each sampling area. Bags were transferred to the cold store and placed in 1 m^3^ plastic containers for the storage experiment, at 0–1°C and 90% relative humidity, in darkness.

### Carrot yield and storage

Carrot yield was calculated individually for each sampling area by dividing the total weight of carrot roots by the area sampled (Table 1).

Carrot storage loss was analysed in mid-March after ~5.5 months of storage. The cold-stored carrots, comprising six 8-kg samples per field, were divided into healthy and diseased carrots based on visual symptoms. Carrot postharvest storability (as a percentage) was calculated as the ratio of the weight of healthy carrots to the total lot weight, multiplied by 100 (Table 1). In March, the mean and median storability percentages were 78% and 82%, respectively. For downstream analyses using categorical explanatory variables (i.e. differential abundance analysis), the samples were divided into two groups: high (>80%) and low (≤80%) storability. The causal pathogens responsible for storage losses were studied and reported by Karisto et al. (2026).

### Soil analysis

Soil analysis and measurement of CEC was performed in the laboratory of Eurofins Finland. The analysis included the sensory analysis of soil type, measurements of electrical conductivity (ISO 11265:1994, modified), pH in water (ISO 10390:2005, modified), macro nutrients determined by the acid ammonium acetate extraction (Vuorinen and Mäkitie 1955), soluble nitrogen (SFS-EN 13654-1:2002), and organic matter (OM%) (SFS-EN 15935:2012). Soil with an OM content of 20% or more was considered to be organic, while soil with an OM content of <20% was considered to be mineral.

### Climate data

To assess daily mean temperature and cumulative rainfall during the growing season, we used 1 × 1 km gridded data from the Finnish Meteorological Institute (Table 1). To simplify the climate parameters, we calculated correlations among them. The mean temperatures during the week, fortnight and month prior to sampling were positively correlated (*r* > 0.8, *P* < 0.05) (Supplementary Fig. S2); therefore, *monthly mean temperature* was selected as the representative parameter. The total rainfall during the growing season was also strongly negatively correlated (*r* = −0.8, *P* < 0.05) with monthly mean temperature (Supplementary Fig. S2). Additional parameters included the mean rainfall during the week before harvest (*preharvest weekly mean rainfall*) and during the month before harvest (*preharvest monthly mean rainfall*).

### DNA extraction and sequencing

Frozen rhizosphere and bulk soil samples were thawed and carefully mixed, and a 250-mg soil subsample was taken with a sterile spatula for DNA extraction using the DNeasy PowerSoil Pro kit (Qiagen). Lysis buffer (800 µl) was added to the samples, followed by brief mixing using a Vortex-Genie 2 (Scientific Industries) and incubation at 65°C for 10 min. After incubation, the samples were disrupted using a TissueLyser II (Qiagen) at 25 Hz for 2 × 5 min and then shaken horizontally in a bead-tube holder (Macherey-Nagel) for 15 min. Finally, the samples were centrifuged for 2 min at 15 000 × *g*, and 600 µl of supernatant was transferred for DNA extraction using a QIAcube Connect, following the manufacturer’s instructions (Qiagen). DNA was eluted in 100 µl of elution buffer. In total, 312 rhizosphere and soil DNA samples were extracted and stored at −20°C.

Amplification of DNA and amplicon sequencing (Illumina MiSeq V3, paired end 2 × 301 bp, 2 × 10 bp dual index, MCS 2.5.0.5/2.6.2.1 and RTA 1.18.54.0) were performed at the Institute of Genomics, University of Tartu, Estonia. For amplification of the V4 region of the bacterial 16S rRNA gene, primers 515 and 806 (Caporaso et al. 2012) were used, and for the fungal internal transcribed spacer region ITS2, primers gITS7 (Ihrmark et al. 2012) and ITS4 (White et al. 1990) were used.

### Bioinformatics

Bioinformatic analyses were performed using PipeCraft 2.0 (Anslan et al. 2017), pipecraft2-manual.readthedocs.io/en/stable, with an OTU workflow that utilizes cutadapt 3.5 (Martin 2011), fqgrep 0.4.4 (Das 2011), and vsearch 2.18.0 (Rognes et al. 2016). Default parameters were used, with the following exceptions: reads were reoriented and primers removed (two mismatches allowed); reads were merged and quality-filtered using a minimum overlap of 15 bp, a minimum length of 150 bp, a maximum of 99 differences, MaxEE = 1 and MaxN = 0. De novo chimera filtering was performed using a threshold of 0.97. In addition, the fungal ITS2 region was extracted from reads using ITSx v1.1.3 (Bengtsson-Palme et al. 2013) and mothur v1.46.1 (Schloss et al. 2009), after which sequence reads were clustered and an OTU table was created using a threshold of 0.97.

OTU sequences were annotated using BLAST v2.11.0+ (Camacho et al. 2009) against the SILVA SSU 138 database (Quast et al. 2012) for bacteria and UNITE (sh_general_release_dynamic_10.05.2021.fasta) (Abarenkov et al. 2021) for fungi, with the following parameters: word size = 7; *e*-value = 0.001; reward = 1; penalty = −1; gap opening = 1; and gap extension = 2. During secondary filtering, OTUs without BLAST results, with <70% identity to bacteria or fungi, or with *e*-values higher than 1 × 10^−24^ were removed. Bacterial OTUs with the same annotation were consolidated. The most abundant bacterial OTUs in the dataset originated from the crop plant: carrot chloroplasts and mitochondria accounted for 115 706 (1.7%) and 30 101 (0.4%) reads, respectively. A further 126 mitochondrial OTUs were identified, comprising 35 928 reads (0.5%) in total. Plant mitochondrial and chloroplast OTUs were excluded from subsequent analyses. For fungi, OTUs with the same *species hypothesis code* (Kõljalg et al. 2013) were consolidated. Finally, OTUs with fewer than five reads were removed. This resulted in 14 947 bacterial and 3 947 fungal OTUs. The median library size per sample was 22 150 reads for the bacterial 16S rRNA gene region (Supplementary Fig. S3) and 16 775 reads for the fungal ITS region (Supplementary Fig. S4). As the smallest library size differed from the median by less than threefold, no samples were removed as outliers. Functional guilds were assigned to fungal taxa using FUNGuild, a tool for fungal ecological guild classification (Nguyen et al. 2016).

### Data analysis and statistics

Alpha-diversity indices (e.g. richness and Shannon diversity estimates) were calculated with the microbiome package (Lahti and Shetty 2019) from rarefied OTU data. OTU data were rarefied using the phyloseq function rarefy_even_depth. For subsequent analyses, such as beta diversity, the raw data were normalized using the geometric mean of pairwise ratios (GMPR) method (Chen et al. 2018), which deals with zero-inflated community data, preserves differences in relative abundances of taxa, and retains more information than rarefaction.

Beta diversity was investigated using non-metric multidimensional scaling (NMDS), the *metaMDS* function and the Bray–Curtis dissimilarity index in the vegan package (Oksanen et al. 2020). The *envfit* function was used to fit environmental parameters to the ordination. Permutational multivariate analysis of variance based on distance matrices was performed using the *adonis2* function and the Bray–Curtis dissimilarity index. The *betadisper* function was used to determine whether the dispersion of multivariate data differed significantly among groups. Core taxa, analysed at 90% prevalence using the microbiome package (Lahti and Shetty 2019), represent OTUs characteristic of a sample type at a given prevalence and detection threshold.

Pearson correlation coefficients between the measured parameters were calculated with cor function, library corrplot (Wei and Simko 2024), using a significance level of 0.05 for all the statistical tests. Factor analysis of mixed data (FAMD) was done with libraries FactoMineR (Lê et al. 2008), vcd, and factoextra (Kassambara and Mundt 2020), to study associations between both quantitative and qualitative variables using principal component analysis for quantitative variables, and multiple correspondence analysis for qualitative variables (Supplementary Figs S7 and  S8).

To study predominance of certain OTUs in high and low storability carrots we cross-correlated the storability with GMPR normalized OTU data input matrices (library microbiome, function associate), using Spearman correlation.

Differential abundance analysis of microbial taxa between experimental groups was performed using the DEseq2 (Love et al. 2014) package in R. DESeq2 utilizes a negative binomial distribution model to detect differences in read counts, accounting for both the mean–variance relationship inherent to count data and overdispersion. The method internally performs normalization to correct for differences in sequencing depth and library composition between samples.

The effects of farming type and green manure use on microbial alpha diversity and richness were studied using a linear mixed-effects model (LMM) fitted with the nlme package (Pinheiro et al. 2023), with field site included as a random factor. Analyses were performed separately for rhizosphere and bulk soil. The full model included farming type, storability, green manure use, and carrot cultivar as explanatory variables. The relationship between storability and green manure use was studied using a separate LMM for the full dataset, with field site as a random factor. Estimated marginal means (EMMs; least-squares means) were computed using the emmeans package from the fitted LMM to obtain model-based means for each factor level and assess differences among treatments. *P*-values were adjusted for multiple testing using Tukey’s honestly significant difference method.

Data were analysed with R 4.4.1 (R Core Team 2024) using the phyloseq package (McMurdie and Holmes 2013) and visualized with ggplot2 (Wickham 2016). Bacterial OTUs are reported at both phylum and family levels, and fungal OTUs are reported at both phylum and genus levels. This is because the barcoding regions and the available databases do not separate lower taxonomic levels well (O’Donnell et al. 2022).

### Data storage and availability

All data will be made available upon publication. Raw sequences, metadata and attributes are available in NCBI GenBank BioProject PRJNA976070 under accessions SAMN35358255–SAMN35358630. Other data are stored in Zenodo: **10.5281/zenodo.17212592**.

## Results

### Soil properties, other environmental factors, carrot storability, and their correlations

Soil properties, management, cultivar, and location information for the studied carrot field sites are presented in Table 1 and Supplementary Fig. S1. In five of the six organically farmed fields, carrots had been grown previously, and all six used green manure in the rotation. In eight of the 20 conventionally managed fields, carrots had not been grown previously, whereas in four fields carrots had been grown within the preceding 3 years (Table 1). Only three conventional field sites, all of which had previously grown carrots, used green manure in the rotation. Soil types ranged from fine sandy till and medium-fine sand to fine clay-rich loam, organic mull and peat (Table 1). Soil types were characterized by distinctive organic matter contents (OM%), ranging from 4% to 80%. Here, OM% was used to describe variation among soil types.

Carrot storability was not associated with OM%, but carrots from the three loamy mineral soils showed the highest storability (Table 1). Soil chemical characteristics were not associated with carrot storability (Supplementary Fig. S2). Soil pH varied among fields from 5 to 7 (Table 1). Soil pH was strongly positively correlated with phosphorus content (*r* = 0.6, *P* < 0.001) and strongly negatively correlated with conductivity (*r* = −0.6, *P* < 0.001), nitrogen content (*r* = −0.7, *P* < 0.001), and OM% (*r* = −0.6, *P* < 0.001). Monthly mean temperature during the growing season was strongly negatively correlated with total rainfall during the growing season (*r* = −0.8, *P* < 0.05) and positively correlated with total carrot yield (*r* = 0.6, *P* < 0.001). Carrot storability was weakly negatively associated with mean rainfall prior to sampling (*r* = −0.3, *P* < 0.01) and positively associated with total rainfall (*r* = 0.3, *P* < 0.05).

Associations among quantitative and qualitative variables were further explored using FAMD, which considers all dependent variables together (Supplementary Figs S5 and S6). FAMD separated the sites according to soil type, management practices (farming type and green manure use), carrot cultivation history, and carrot cultivar. The first dimension separated sites according to conductivity, soil type (mineral vs. organic), pH, bacterial richness, sulphur content, CEC, OM%, farming type (conventional or organic), and carrot yield. Sites with organic soils had higher conductivity, CEC, OM%, sulphur and nitrogen contents, and lower pH (5.7). In contrast, sites with mineral soils had a higher pH (6.5; Table 1). The second dimension separated sites according to mean rainfall during the week preceding harvest, phosphorus content, carrot cultivar, and cultivation history (Supplementary Fig. S5). Sites with high phosphorus levels, the Newhall carrot cultivar, and rainy weather clustered together. In the FAMD analysis, carrot storability was collinear with the second dimension but contributed little to it. Thus, carrot storability was only weakly associated with the parameters contributing to the second dimension. Moreover, green manure use was not significantly associated with storability (LMM *F*_1,24_ = 1.25, *P* > 0.27).

### Microbial community characteristics and associations with environmental factors and carrot storability

#### Microbial community composition, diversity and species richness

##### The main microbial phyla in rhizosphere and bulk soil

**Figure 1:**
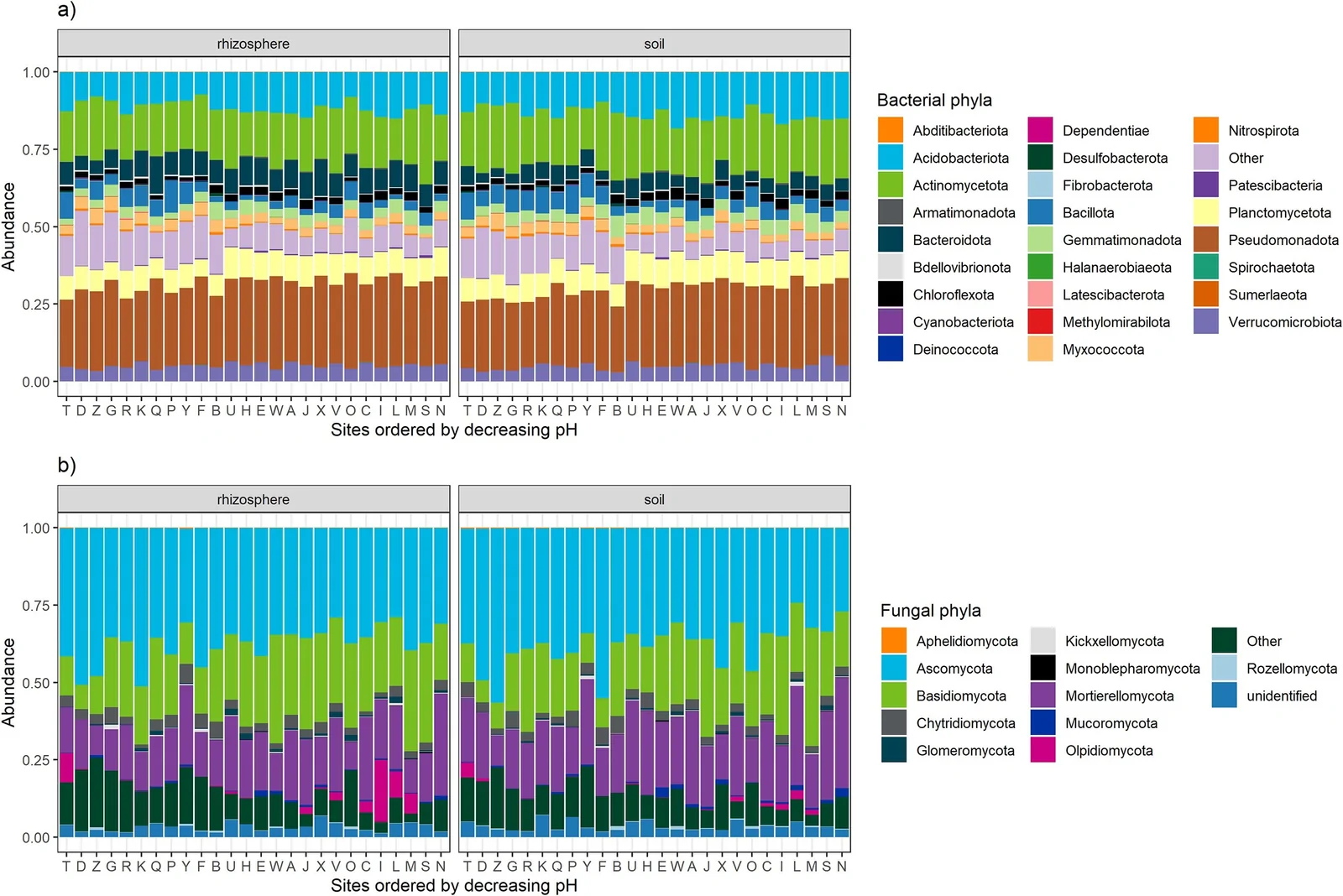
Relative abundance of main bacterial and fungal phyla. *X*-axis sites ordered by decreasing pH. The figure displays the dominant bacterial phyla across sample types and sites, with rare phyla grouped into the category ‘Other’ for clarity. Colours represent different phyla as indicated in the legend. Data are presented as relative proportions.

Based on the bacterial 16S rRNA gene sequence data, the most abundant bacterial phyla in the rhizosphere and bulk soil samples, respectively, were *Pseudomonadota* (26%, 24%), *Actinomycetota* (17%, 19%), and *Acidobacteriota* (13%, 15%) (Fig. 1a; Oren et al. 2021, 2022). Based on the ITS2 sequence data, the most abundant fungal phyla in both the rhizosphere and the bulk soil communities were *Ascomycota* (42%, 41%), *Basidiomycota* (28%, 24%), and *Mortierellomycota* (18%, 23%) (Fig. 1b).

##### Microbial diversity in rhizosphere and bulk soil

All bacterial and fungal OTUs were present in both rhizosphere and bulk soil samples. The bacterial dataset comprised 14 947 raw OTUs, which were reduced to 14 821 OTUs after rarefaction. The fungal dataset included 3947 raw OTUs, with 3803 OTUs retained following rarefaction. The mean observed bacterial OTU richness in a sample was significantly higher in the rhizosphere than in the bulk soil (*P* < 0.001), 2220 and 2154 OTUs respectively (Table 1, Supplementary Fig. S7). The lowest bacterial OTU richness, 1523, was found in the conventionally managed field S with acidic organic bulk soil (low pH, high nitrogen content, Table 1), and the highest, 2853, in the organically farmed field Y with mineral bulk soil (high pH, low nutrients) (Table 1). The median observed fungal OTU richness was similar in rhizosphere and in bulk soil, 321 and 330, respectively (*P* = 0.3) (Supplementary Fig. S7). The lowest fungal OTU richness was 203, found in conventionally managed organic rhizosphere soil (field M, low pH, low nutrients) and the highest, 476, was in the organically farmed mineral rhizosphere soil (field Y, high pH, low nutrients) (Table 1).

**Figure 2:**
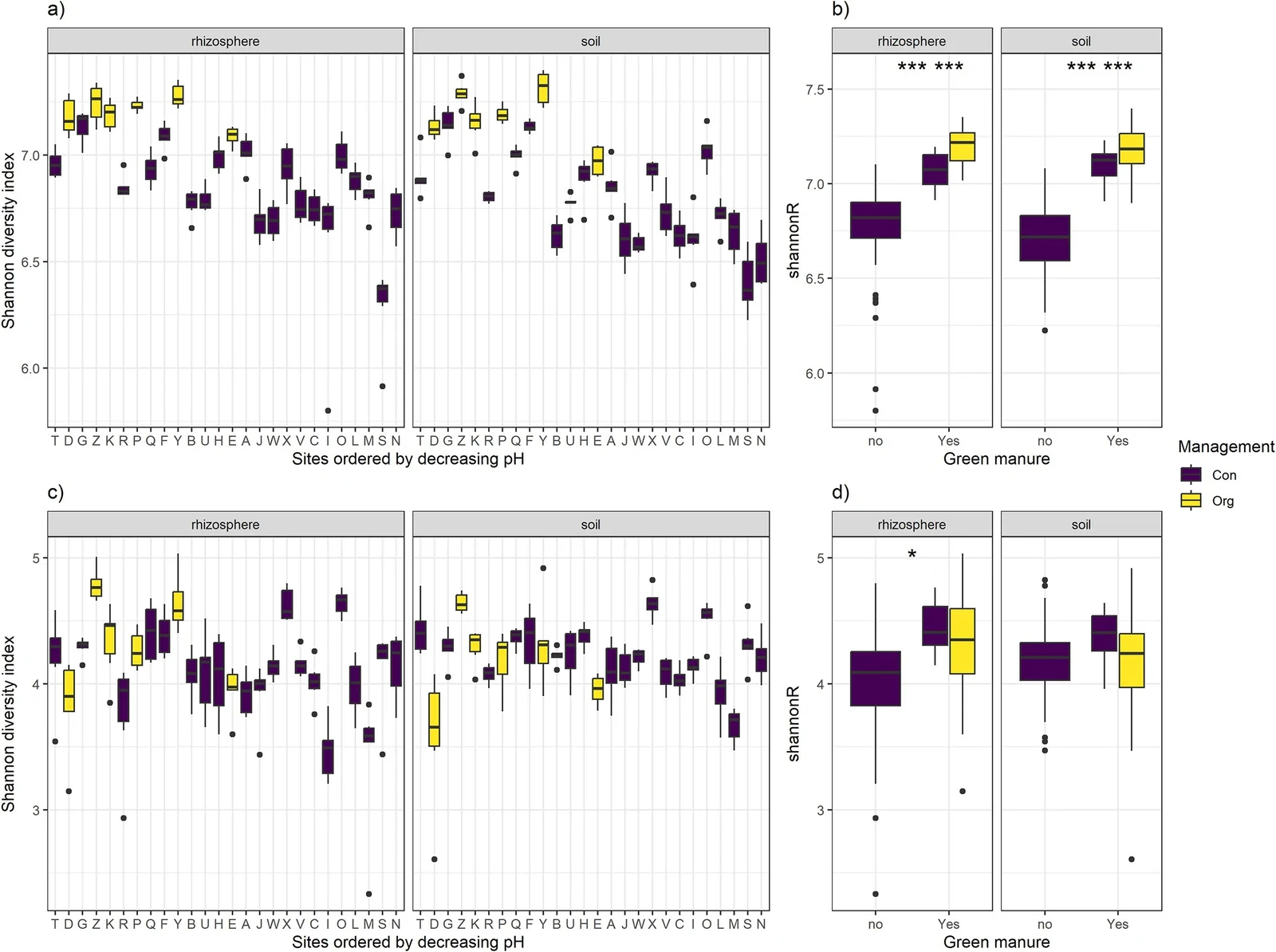
Shannon diversity indices of bacterial (a, b) and fungal (c, d) OTUs across different sample types. Boxplots represent the median and interquartile range. Diversity values (a, c) grouped by sites, ordered by decreasing soil pH. Diversity values (b, d) grouped by green manure use and farming type. According to the LMM, there were statistically significant differences between (b) bacterial diversity in both farming type and green manure use *P* < 0.001**, and (d) fungal diversity between sites that use green manure *P* < 0.01.

Mean bacterial Shannon diversity was 6.9 and was higher in rhizosphere than in bulk soil samples (*P* < 0.001). It was lowest in conventionally managed acidic organic soil (field S) and highest in organically farmed mineral soils (fields Y and Z) with high pH and low nitrogen content (Table 1, Fig. 2a). Soil pH and bacterial alpha diversity were positively correlated; sites with higher pH values in mineral soils also had greater bacterial diversity (*r* > 0.6, *P* < 0.001; Supplementary Fig. S2). Both bacterial diversity and observed OTU richness were negatively correlated with soil conductivity, sulphur content and CEC (*r* < −0.5, *P* < 0.001; Supplementary Fig. S2).

Farming type and use of green manure significantly influenced bacterial OTU richness and Shannon diversity. Bacterial OTU richness was statistically significantly higher in organic than in conventional farms in both the rhizosphere and bulk soil (LMM rhizosphere: *F*₁,₂₀ = 43.08, *P* < 0.001; bulk soil: *F*₁,₂₀ = 46.87, *P* < 0.001), as was Shannon diversity (rhizosphere: *F*₁,₂₀ = 25.74, *P* < 0.001; bulk soil: *F*₁,₂₀ = 32.33, *P* < 0.001) (Fig. 2b, Supplementary Fig. S7). Likewise, the inclusion of green manure in the rotation increased both bacterial richness (LMM rhizosphere: *F*₁,₂₀ = 8.07, *P* = 0.01; bulk soil: *F*₁,₂₀ = 15.85, *P* < 0.001) and diversity (rhizosphere: *F*₁,₂₀ = 17.90, *P* < 0.001; bulk soil: *F*₁,₂₀ = 23.48, *P* < 0.001). In contrast, storability and cultivar had no significant effects (*P* > 0.05). Tukey-adjusted EMMs no differences between farming types (*P* > 0.3), but significantly higher values with green manure compared to no green manure (*P* < 0.01) (Fig. 2b, Supplementary Fig. S7).

Mean fungal Shannon diversity was 4.1 in rhizosphere samples and 4.2 in bulk soil samples (*P* > 0.1). It was lowest in conventionally managed acidic organic rhizosphere soil (field M) and highest in organically farmed mineral rhizosphere soil (field Z; high pH and low nutrient levels) (Table 1, Fig. 2c). There was no strong linear association between soil physicochemical parameters and fungal diversity. Fungal Shannon diversity and OTU richness were negatively correlated (*r* < −0.3, *P* < 0.001) with temperature and rainfall: diversity was higher at sites with lower temperatures and less rain preceding harvest (Supplementary Figs S2 and S5). Green manure use significantly increased fungal Shannon diversity in rhizosphere soil (LMM *F*_1,20_ = 5.13, *P* = 0.03) (Fig. 2d, Supplementary Fig. S8). In contrast, farming type, storability and cultivar had no significant effects (*P* > 0.05). Tukey-adjusted EMMs confirmed significantly higher diversity in fields with green manure than in those without (*P* < 0.05).

##### Core taxa and drivers of community composition in rhizosphere and bulk soil

The core bacterial and fungal OTUs in both the rhizosphere and the bulk soil were representative of the main phyla and were mainly shared (324 bacterial OTUs and 67 fungal OTUs) between both sample types (Fig. 1, Supplementary Table 1). In addition, 72 bacterial OTUs were characteristic to only rhizosphere and 38 to bulk soil. There were 57 fungal core OTUs found in rhizosphere and 56 in bulk soil, from which 46 were shared between the sample types (Supplementary Table 1).

**Figure 3:**
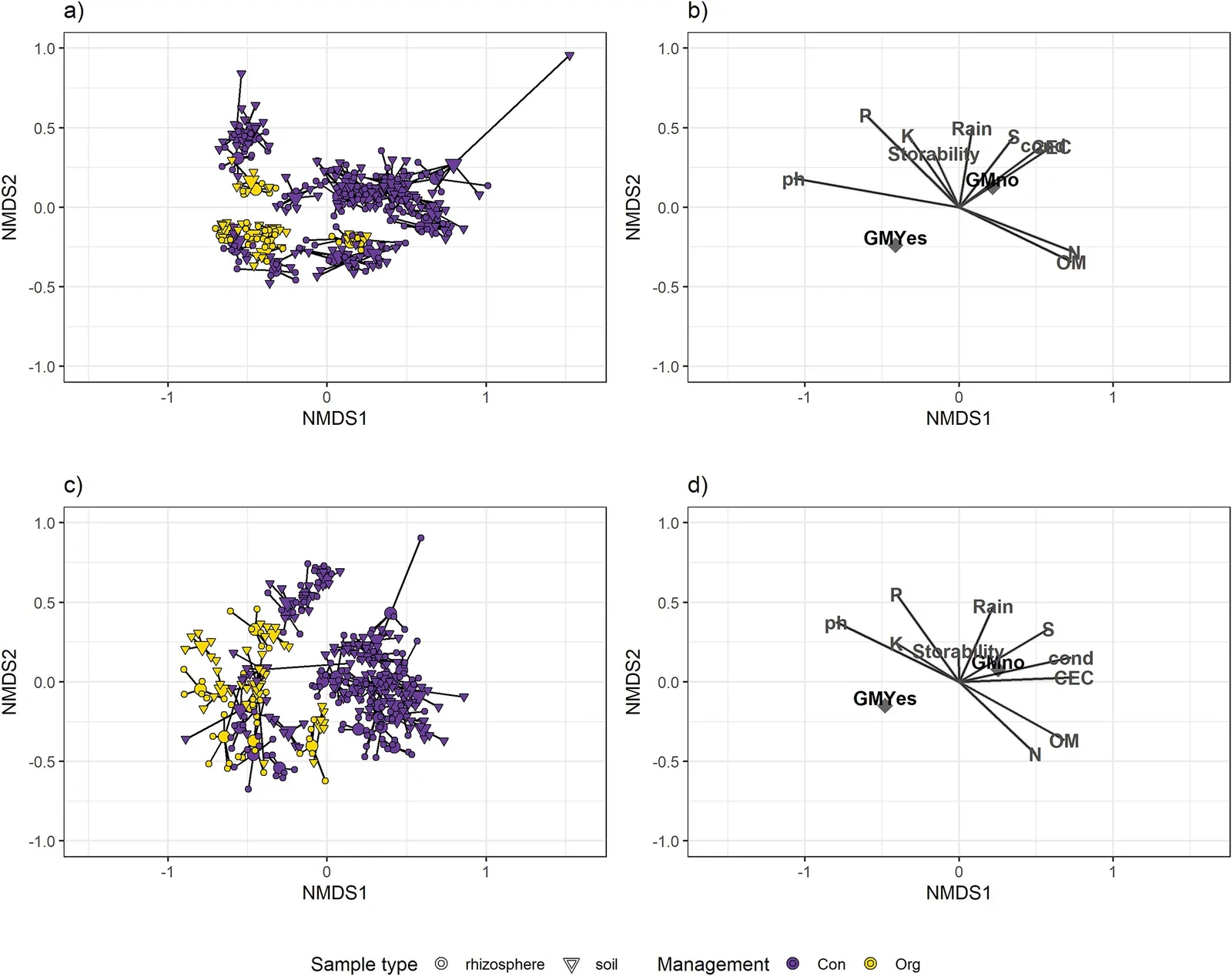
A 2D NMDS of (a) bacterial and (c) fungal community highlighting the sites, sample types (° rhizosphere, Δ bulk soil) and farming [conventional (purple) vs. organic (yellow)], and showing the soil characteristics (OM%, pH, P, K, S, N, CEC, rain, storability, green manure, and conductivity) and carrot storability fitted as vectors in the same ordination for (b) bacteria and (d) fungi. Environmental factors unit length vectors are scaled by their correlation (*P* < 0.05).

Both bacterial and fungal communities grouped according to the field, and the rhizosphere and bulk soil samples of each site shared similar microbial communities (Fig. 3a–c). The fields with high OM% and low pH clustered tightly together, and so did the mineral field sites with a higher pH. Thus, the soil type and its main chemical characteristics pH, OM%, and phosphorus and nitrogen content appear to be the primary drivers of bacterial and fungal communities, respectively, along the first axis (Fig. 3a–d, Supplementary Table 2). Variation in *preharvest week mean of rainfall*, soil CEC, conductivity, and other macronutrients K and S, as well as the use of green manure, especially for fungi, aligned with the 2nd axis and significantly correlated with the ordination (Figs 3a–d, Supplementary Table 2). Furthermore, the organic farm sites clustered tightly together and shared similarities especially in their bacterial communities. Carrot storability, aligned with the 2nd axis, had a weak but significant correlation with the NMDS ordination for both bacteria and fungi (Supplementary Table 2).

Field site was the dominant factor explaining variation in both bacterial and fungal community composition (bacteria: *R*² = 0.63 and 0.68; fungi: *R*² = 0.63 and 0.68; PERMANOVA, *P* < 0.001). When field was used as a grouping factor, the marginal effects of the remaining parameters were evaluated separately for rhizosphere and bulk soil; however, none was statistically significant (Supplementary Table 3). Dispersion differed significantly among fields; therefore, the analysis may have slightly overestimated the field effect.

#### Rhizosphere and bulk soil microbes associated with carrot storability

##### Differential abundance analysis—indicators of high and low storability

The average bacterial Shannon diversity was 6.9 in the rhizosphere and bulk soil of carrots, regardless of whether they exhibited low or high storability (LMM *P* > 0.2). Similarly, observed bacterial OTU richness was nearly identical between low- and high-storability carrots, with values of 2247 and 2196 in the rhizosphere, and 2171 and 2139 in bulk soil (LMM *P* > 0.3).

**Figure 4:**
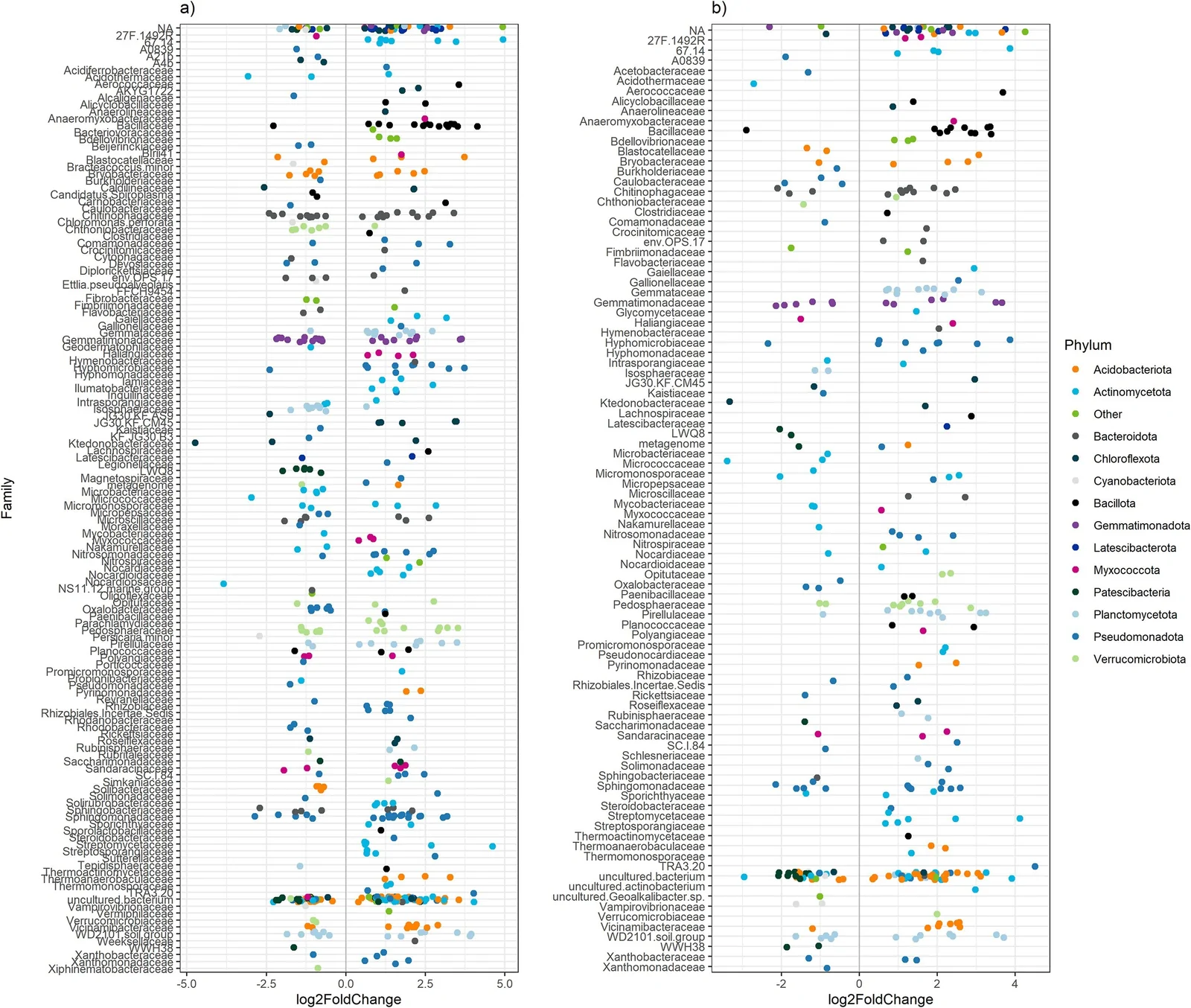
Differential abundance analysis (*P* < 0.05) of indicator bacterial families in (a) rhizosphere and (b) bulk soil.

Differential abundance analysis comparing microbial communities associated with high (>80%) and low (≤80%) carrot storability identified 356 and 239 bacterial OTUs in rhizosphere and bulk soil samples, respectively, that were associated with high storability (Fig. 4). Bacteria most strongly associated with high storability included taxa belonging to *Streptomycetaceae* and *Solirubrobacterales* (*Actinomycetota*), *Bacillaceae* and *Aerococcaceae* (*Bacillota*), *Hyphomicrobiaceae* and *TRA3–20* (*Pseudomonadota*), *Gemmatimonadaceae* (*Gemmatimonadota*), and *Tepidisphaerales* WD2101 (*Planctomycetota*). In contrast, 103 and 228 bacterial OTUs in rhizosphere and bulk soil samples, respectively, were associated with low storability, including uncultured *Ktedonobacteraceae* (*Chloroflexota*) and *Micrococcaceae* (*Actinomycetota*) (Fig. 4).

In rhizosphere samples, both observed richness (306 vs. 344) and Shannon diversity (4.0 vs. 4.2) were significantly lower in low-storability carrots compared with high-storability carrots (LMM richness: *F*₁,₂₀ = 6.00, *P* = 0.024; Tukey-adjusted EMM contrast: *P* = 0.014; Shannon diversity: *F*₁,₂₀ = 5.63, *P* = 0.028; Tukey-adjusted EMM contrast: *P* = 0.018). In bulk soil, the differences were smaller and not significantly different, with richness values of 338 vs. 321 and Shannon diversity of 4.3 vs. 4.1.

**Figure 5:**
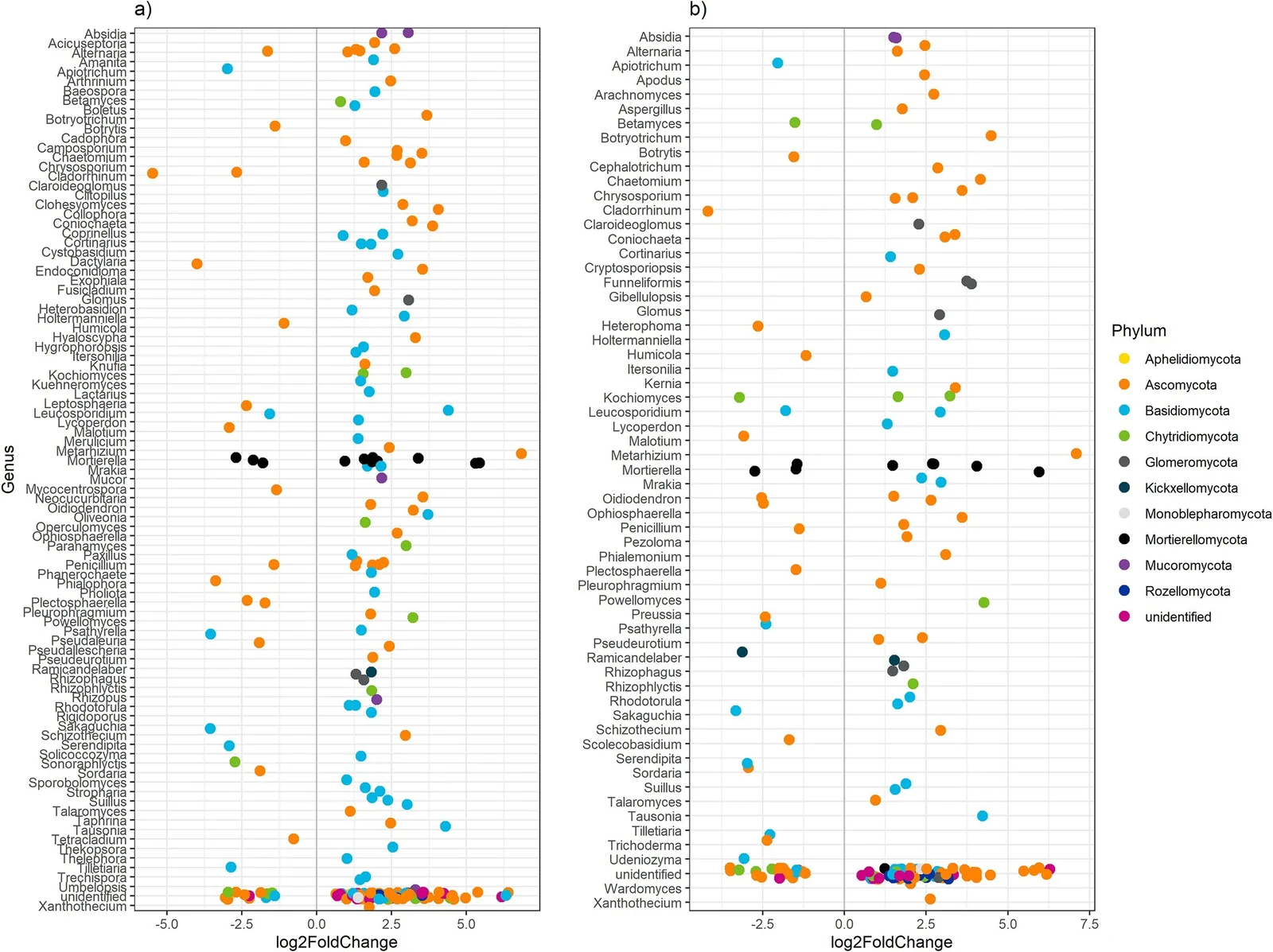
Differential abundance analysis (*P* < 0.05) of indicator fungal genera in (a) rhizosphere and (b) bulk soil. Many more fungal OTUs were associated with high than with low storability.

Differential abundance analysis identified 185 and 121 fungal OTUs indicative of high storability in rhizosphere and bulk soil, respectively. The taxa most strongly indicative (log fold change > 4) of high storability included the ascomycetes *Metarhizium* sp., unidentified *Pleosporales* and *Microascales, Botryotrichum* sp. and *Chaetomium* sp.; *Tausonia* sp. (*Basidiomycota*); *Powellomyces* sp. (*Chytridiomycota*); and *Mortierella* sp. (*Mortierellomycota*). Furthermore, 38 and 47 fungal OTUs were indicative of low storability in rhizosphere and bulk soil, respectively. Differential abundance analysis showed that the ascomycete *Cladorrhinum* sp. was most indicative of low storability in both rhizosphere and bulk soil (Fig. 5; see also fungal OTUs potentially assigned as endophytes in Supplementary Table 4).

##### Microbial taxa abundance as a predictor of carrot storability

In the rhizosphere bacteriome, high carrot storability correlated positively (*r* > 0.5, *P* < 0.001) with abundancy of OTUs belonging to Actinomycetota, Acidobacteriota (*Vicinamibacteraceae, Thermoanaerobaculaceae*), Bacillota (*Bacillaceae*), RCP2-54, Planctomycetota (*Gemmataceae*, WD2101), and Verrucomicrobiota (*Pedosphaeraceae*) (Table 2). In the bulk soil bacteriome, high storability correlated positively with abundance of Actinomycetota (unknown 0319–7L14), Planctomycetota (unknown Gemmatales and Planctomycetales, *Rubinisphaeraceae*), Bacillota (*Planococcaceae*), and Acidobacteriota (unknown Vicinamibacterales)(Table 2). Low carrot storability correlated (*r* < −0.5, *P* < 0.001) with the presence of Pseudomonadota Burkholderiales agricultural soil bacterium SC-I-84 in both the rhizosphere and bulk soil (Table 2).

**Table 2:** Correlation analysis between carrot storability and bacterial OTU abundance based on GMPR-normalized data. Spearman correlation coefficients calculated separately for rhizosphere and bulk soil. Table shows moderate and strong correlations |>0.35|, *P*<0.05.

Phylum	Class	Family	Genus	rhizosphere	soil
Acidobacteriota	Acidobacteriae	Bryobacteraceae	Bryobacter	0.42	
Acidobacteriota	Acidobacteriae	Bryobacteraceae	Bryobacter	0.43	0.48
Acidobacteriota	Acidobacteriae	metagenome	NA	0.46	0.48
Acidobacteriota	Blastocatellia	Blastocatellaceae	OLB17	0.39	0.42
Acidobacteriota	Blastocatellia	uncultured.bacterium	NA	-0.42	
Acidobacteriota	Subgroup.22	NA	NA	0.4	0.41
Acidobacteriota	Subgroup.22	NA	NA	0.42	0.42
Acidobacteriota	Subgroup.25	NA	NA	0.43	0.39
Acidobacteriota	Subgroup.5	NA	NA	0.38	0.43
Acidobacteriota	Thermoanaerobaculia	Thermoanaerobaculaceae	Subgroup.10	0.49	0.51
Acidobacteriota	Vicinamibacteria	uncultured	uncultured.Acidobacteria.bacterium	0.42	0.46
Acidobacteriota	Vicinamibacteria	uncultured	uncultured.Acidobacterium.sp.	0.4	0.4
Acidobacteriota	Vicinamibacteria	uncultured	uncultured.bacterium	0.43	0.45
Acidobacteriota	Vicinamibacteria	uncultured	uncultured.bacterium	0.51	0.5
Acidobacteriota	Vicinamibacteria	uncultured	uncultured.bacterium		0.42
Acidobacteriota	Vicinamibacteria	Vicinamibacteraceae	uncultured.Acidobacteria.bacterium	0.41	
Acidobacteriota	Vicinamibacteria	Vicinamibacteraceae	uncultured.Acidobacteria.bacterium	0.43	0.47
Acidobacteriota	Vicinamibacteria	Vicinamibacteraceae	uncultured.bacterium	0.4	0.52
Acidobacteriota	Vicinamibacteria	Vicinamibacteraceae	uncultured.bacterium	0.44	0.43
Acidobacteriota	Vicinamibacteria	Vicinamibacteraceae	uncultured.bacterium		0.4
Acidobacteriota	Vicinamibacteria	Vicinamibacteraceae	uncultured.bacterium		0.44
Actinomycetota syn. Actinobacteriota	Acidimicrobiia	uncultured	uncultured.bacterium	0.39	0.46
Actinomycetota syn. Actinobacteriota	Acidimicrobiia	Iamiaceae	Iamia	0.46	0.41
Actinomycetota syn. Actinobacteriota	Acidimicrobiia	uncultured	uncultured.bacterium		0.45
Actinomycetota syn. Actinobacteriota	Actinobacteria	uncultured.actinobacterium	NA	0.51	0.43
Actinomycetota syn. Actinobacteriota	Actinobacteria	uncultured.Actinomycetales.bacterium	NA	0.44	0.39
Actinomycetota syn. Actinobacteriota	Actinobacteria	uncultured.bacterium	NA	0.53	0.56
Actinomycetota syn. Actinobacteriota	Actinobacteria	uncultured.bacterium	NA	0.55	0.5
Actinomycetota syn. Actinobacteriota	Actinobacteria	Mycobacteriaceae	Mycobacterium	-0.41	-0.48
Actinomycetota syn. Actinobacteriota	Actinobacteria	Intrasporangiaceae	Pedococcus.Phycicoccus	-0.42	-0.37
Actinomycetota syn. Actinobacteriota	Actinobacteria	Intrasporangiaceae	Terrabacter	-0.37	-0.4
Actinomycetota syn. Actinobacteriota	Actinobacteria	Nocardioidaceae	Nocardioides		0.41
Actinomycetota syn. Actinobacteriota	Actinobacteria	Pseudonocardiaceae	Amycolatopsis	0.39	0.45
Actinomycetota syn. Actinobacteriota	Actinobacteria	Streptomycetaceae	Streptomyces	0.38	0.4
Actinomycetota syn. Actinobacteriota	Thermoleophilia	Gaiellaceae	Gaiella	0.41	0.47
Actinomycetota syn. Actinobacteriota	Thermoleophilia	uncultured	metagenome		0.41
Actinomycetota syn. Actinobacteriota	Thermoleophilia	67.14	uncultured.bacterium	0.39	0.5
Actinomycetota syn. Actinobacteriota	Thermoleophilia	67.14	uncultured.bacterium		0.48
Actinomycetota syn. Actinobacteriota	Thermoleophilia	Solirubrobacteraceae	Conexibacter	-0.4	-0.37
Actinomycetota syn. Actinobacteriota	Thermoleophilia	uncultured.bacterium	NA		0.42
Bacteroidota	Bacteroidia	Chitinophagaceae	Ferruginibacter	0.4	0.46
Bacteroidota	Bacteroidia	Chitinophagaceae	Flavisolibacter	0.4	0.4
Bacteroidota	Bacteroidia	Chitinophagaceae	Flavisolibacter	0.43	0.43
Bacteroidota	Bacteroidia	Chitinophagaceae	Terrimonas	0.45	0.47
Bacteroidota	Bacteroidia	Chitinophagaceae	uncultured	0.43	
Bacteroidota	Bacteroidia	Chitinophagaceae	uncultured		-0.42
Bacteroidota	Bacteroidia	Chitinophagaceae	uncultured		-0.4
Bacteroidota	Bacteroidia	Saprospiraceae	uncultured	0.44	0.39
Bacteroidota	Bacteroidia	Hymenobacteraceae	Adhaeribacter	0.36	0.41
Bacteroidota	Bacteroidia	Crocinitomicaceae	Fluviicola		0.42
Chloroflexota syn. Chloroflexi	Anaerolineae	Anaerolineaceae	uncultured		0.4
Chloroflexota syn. Chloroflexi	Anaerolineae	Anaerolineaceae	uncultured		0.42
Chloroflexota syn. Chloroflexi	Anaerolineae	A4b	uncultured.bacterium		0.46
Chloroflexota syn. Chloroflexi	Anaerolineae	uncultured.bacterium	NA		0.42
Chloroflexota syn. Chloroflexi	Chloroflexia	JG30.KF.CM45	uncultured.bacterium	0.43	0.47
Chloroflexota syn. Chloroflexi	Dehalococcoidia	metagenome	NA		0.41
Chloroflexota syn. Chloroflexi	JG30.KF.CM66	NA	NA	0.41	0.43
Chloroflexota syn. Chloroflexi	JG30.KF.CM66	NA	NA		0.41
Chloroflexota syn. Chloroflexi	KD4.96	NA	NA	-0.47	-0.36
Chloroflexota syn. Chloroflexi	OLB14	NA	NA	0.38	0.42
Chloroflexota syn. Chloroflexi	OLB14	NA	NA	0.42	0.41
Cyanobacteriota syn. Cyanobacteria	Cyanobacteriia	uncultured.marine.eukaryote	NA		-0.43
Bacillota syn. Firmicutes	Bacilli	Bacillaceae	Bacillus		0.44
Bacillota syn. Firmicutes	Bacilli	Bacillaceae	Pseudogracilibacillus	0.38	0.43
Bacillota syn. Firmicutes	Bacilli	Bacillaceae	Pseudogracilibacillus	0.4	0.41
Bacillota syn. Firmicutes	Bacilli	Bacillaceae	Pseudogracilibacillus	0.5	0.53
Bacillota syn. Firmicutes	Bacilli	Bacillaceae	uncultured	0.37	0.42
Bacillota syn. Firmicutes	Bacilli	Bacillaceae	uncultured	0.41	0.44
Bacillota syn. Firmicutes	Bacilli	Bacillaceae	uncultured	0.47	0.39
Bacillota syn. Firmicutes	Bacilli	Bacillaceae	uncultured	0.48	0.53
Bacillota syn. Firmicutes	Bacilli	Bacillaceae	uncultured		0.42
Bacillota syn. Firmicutes	Bacilli	Bacillaceae	Virgibacillus	0.38	0.41
Bacillota syn. Firmicutes	Bacilli	Bacillaceae	Virgibacillus.soli		0.4
Bacillota syn. Firmicutes	Bacilli	Planococcaceae	Sporosarcina	0.53	0.41
Bacillota syn. Firmicutes	Bacilli	Aerococcaceae	Aerococcus	0.48	0.39
Bacillota syn. Firmicutes	Bacilli	Paenibacillaceae	Ammoniphilus	0.42	0.42
Gemmatimonadota	BD2.11.terrestrial.group	NA	NA	0.45	0.4
Gemmatimonadota	BD2.11.terrestrial.group	NA	NA	0.41	0.38
Gemmatimonadota	BD2.11.terrestrial.group	NA	NA		0.42
Gemmatimonadota	Gemmatimonadetes	Gemmatimonadaceae	uncultured	-0.41	
Gemmatimonadota	Gemmatimonadetes	Gemmatimonadaceae	uncultured	0.36	0.41
Gemmatimonadota	Gemmatimonadetes	Gemmatimonadaceae	uncultured	0.45	0.45
Gemmatimonadota	Gemmatimonadetes	Gemmatimonadaceae	uncultured	0.47	0.48
Gemmatimonadota	Gemmatimonadetes	Gemmatimonadaceae	uncultured		0.4
Gemmatimonadota	Gemmatimonadetes	Gemmatimonadaceae	uncultured		0.41
Gemmatimonadota	Gemmatimonadetes	Gemmatimonadaceae	uncultured		0.42
Gemmatimonadota	S0134.terrestrial.group	NA	NA	0.46	0.43
Latescibacterota	uncultured.bacterium	NA	NA	0.4	0.41
Myxococcota	Polyangia	Haliangiaceae	Haliangium	0.41	0.39
Myxococcota	Polyangia	Sandaracinaceae	Sandaracinus	0.41	
Myxococcota	Polyangia	Sandaracinaceae	uncultured		-0.41
Nitrospirota	Nitrospiria	Nitrospiraceae	Nitrospira		0.41
Patescibacteria	Saccharimonadia	uncultured.bacterium	NA		-0.41
Patescibacteria	Saccharimonadia	WWH38	uncultured.bacterium	-0.42	
Patescibacteria	Saccharimonadia	WWH38	uncultured.bacterium		-0.42
Planctomycetota	OM190	NA	NA	0.45	
Planctomycetota	Phycisphaerae	uncultured.bacterium	NA	0.42	
Planctomycetota	Phycisphaerae	WD2101.soil.group	uncultured.bacterium	-0.43	-0.42
Planctomycetota	Phycisphaerae	WD2101.soil.group	uncultured.bacterium	-0.42	-0.46
Planctomycetota	Phycisphaerae	WD2101.soil.group	uncultured.bacterium	0.44	0.44
Planctomycetota	Phycisphaerae	WD2101.soil.group	uncultured.bacterium	0.5	0.47
Planctomycetota	Phycisphaerae	WD2101.soil.group	uncultured.planctomycete	0.48	0.55
Planctomycetota	Phycisphaerae	WD2101.soil.group	uncultured.soil.bacterium	0.41	
Planctomycetota	Phycisphaerae	WD2101.soil.group	uncultured.soil.bacterium	0.41	0.38
Planctomycetota	Planctomycetes	Gemmataceae	Gemmata	0.55	0.51
Planctomycetota	Planctomycetes	Gemmataceae	uncultured	0.45	
Planctomycetota	Planctomycetes	Pirellulaceae	Pirellula	0.4	
Planctomycetota	Planctomycetes	Pirellulaceae	Pirellula	0.43	0.49
Planctomycetota	Planctomycetes	Pirellulaceae	Pirellula	0.45	0.43
Planctomycetota	Planctomycetes	Pirellulaceae	Pirellula		0.4
Planctomycetota	Planctomycetes	Rubinisphaeraceae	SH.PL14	0.37	0.44
Planctomycetota	Planctomycetes	Rubinisphaeraceae	uncultured	0.51	
Planctomycetota	Planctomycetes	uncultured	uncultured.bacterium	-0.43	
Planctomycetota	Planctomycetes	uncultured	uncultured.Planctomycetales.bacterium	0.52	0.43
Planctomycetota	Planctomycetes	uncultured	uncultured.planctomycete	0.43	0.44
Planctomycetota	vadinHA49	NA	NA		-0.42
Pseudomonadota syn. Proteobacteria	Alphaproteobacteria	Hyphomonadaceae	Hirschia	0.41	
Pseudomonadota syn. Proteobacteria	Alphaproteobacteria	uncultured	uncultured.Alphaproteobacteria.bacterium	-0.41	
Pseudomonadota syn. Proteobacteria	Alphaproteobacteria	Hyphomicrobiaceae	Pedomicrobium	0.48	0.47
Pseudomonadota syn. Proteobacteria	Alphaproteobacteria	Hyphomicrobiaceae	Pedomicrobium		0.43
Pseudomonadota syn. Proteobacteria	Alphaproteobacteria	Methyloligellaceae	uncultured	-0.39	-0.42
Pseudomonadota syn. Proteobacteria	Alphaproteobacteria	uncultured	uncultured.Alphaproteobacteria.bacterium		0.44
Pseudomonadota syn. Proteobacteria	Alphaproteobacteria	Sphingomonadaceae	Sphingomonas		0.43
Pseudomonadota syn. Proteobacteria	Alphaproteobacteria	uncultured.bacterium	NA	0.39	0.41
Pseudomonadota syn. Proteobacteria	Gammaproteobacteria	Burkholderiaceae	Burkholderia.Caballeronia.Paraburkholderia	-0.37	-0.48
Pseudomonadota syn. Proteobacteria	Gammaproteobacteria	Comamonadaceae	Ramlibacter	-0.48	-0.37
Pseudomonadota syn. Proteobacteria	Gammaproteobacteria	Comamonadaceae	Variovorax		0.43
Pseudomonadota syn. Proteobacteria	Gammaproteobacteria	Nitrosomonadaceae	MND1	0.45	0.41
Pseudomonadota syn. Proteobacteria	Gammaproteobacteria	Nitrosomonadaceae	MND1	0.45	
Pseudomonadota syn. Proteobacteria	Gammaproteobacteria	SC.I.84	uncultured.bacterium	-0.54	-0.54
Pseudomonadota syn. Proteobacteria	Gammaproteobacteria	TRA3.20	uncultured.bacterium	0.4	0.38
Pseudomonadota syn. Proteobacteria	Gammaproteobacteria	TRA3.20	uncultured.beta.proteobacterium	0.42	0.37
Pseudomonadota syn. Proteobacteria	Gammaproteobacteria	Unknown.Family	uncultured		0.42
Pseudomonadota syn. Proteobacteria	Gammaproteobacteria	Rhodanobacteraceae	Ahniella	0.48	0.41
RCP2.54	uncultured.bacterium	NA	NA	0.5	0.58
Verrucomicrobiota	Verrucomicrobiae	Chthoniobacteraceae	Chthoniobacter		-0.41
Verrucomicrobiota	Verrucomicrobiae	Pedosphaeraceae	uncultured.bacterium	0.39	0.52
Verrucomicrobiota	Verrucomicrobiae	Pedosphaeraceae	uncultured.bacterium	0.4	0.45
Verrucomicrobiota	Verrucomicrobiae	Pedosphaeraceae	uncultured.soil.bacterium	0.39	0.4
Verrucomicrobiota	Verrucomicrobiae	Verrucomicrobiaceae	uncultured	0.46	

The abundance of ascomycetous fungal OTUs representing Ascomycetous *Chrysosporium* sp., *Metarhizium* sp. and *Ophiosphaerella* sp., Mortierellomycotan *Mortierella* sp., and an unknown Basidiomycota OTU correlated positively (*r* > 0.5, *P* < 0.001) with high carrot storability, in both rhizosphere and bulk soil (Table 3). Several Rozellomycota, and Glomeromycotan arbuscular mycorrhizal OTUs also had a positive correlation with storability (*r >* 0.35, *P* < 0.001, Table 3). Low carrot storability was moderately (*r* < *−*0.35, *P* < 0.001) associated with the presence of several Ascomycetous *Mycocentrospora* sp., *Didymella* sp., unidentified *Pleosporales, Scolecobasidium* sp., *Glarea* sp., unidentified *Coniochaetales, Plectosphaerella* sp., unidentified *Nectriaceae*, and *Cladorrhinum* sp., Basidiomycotan *Cutaneotrichosporon* sp., Chytridiomycotan unidentified *Spizellomycetales*, and Mortierellomycotan *Mortierella* sp. (Table 3).

**Table 3:** Correlation analysis between carrot storability and fungal OTU abundance based on GMPR-normalized data. Spearman correlation coefficients calculated separately for rhizosphere and bulk soil. Table shows moderate and strong correlations |>0.35|, *P*<0.05.

Phylum	Genus	Species	rhizosphere	soil
Ascom	Arachnomyces	sp	0.39	
Ascom	Ascomycota	sp	0.45	0.46
Ascom	Cephalotrichum	columnare	0.45	0.39
Ascom	Chrysosporium	pseudomerdarium	0.47	0.52
Ascom	Chrysosporium	sp	0.45	0.54
Ascom	Cistella	sp	0.48	
Ascom	Cladorrhinum	sp	-0.4	-0.39
Ascom	Coniochaeta	sp	0.4	0.4
Ascom	Crocicreas	pallidum	0.39	
Ascom	Didymella	sancta	-0.39	-0.39
Ascom	Glarea	sp		-0.37
Ascom	Helicosporium	aquaticum	0.38	
Ascom	Helotiales	sp		0.39
Ascom	Kernia	sp	0.48	0.48
Ascom	Lasiosphaeriaceae	sp		0.39
Ascom	Leucosphaerina	arxii	0.39	0.44
Ascom	Metapochonia	suchlasporia		0.42
Ascom	Metarhizium	marquandii	0.62	0.58
Ascom	Microascales	sp	0.45	0.45
Ascom	Mycocentrospora	acerina		-0.38
Ascom	Nectriaceae	sp	-0.37	-0.39
Ascom	Oidiodendron	sp	0.4	0.42
Ascom	Ophiosphaerella	sp	0.52	0.47
Ascom	Penicillium	solitum	0.38	
Ascom	Plectosphaerella	cucumerina	-0.37	
Ascom	Pleosporales	sp	0.36	
Ascom	Pleosporales	sp	0.44	0.44
Ascom	Pleosporales	sp	-0.37	
Ascom	Pleosporales	sp	0.44	
Ascom	Scolecobasidium	constrictum	-0.43	
Basid	Agaricomycetes	sp	0.46	0.54
Basid	Basidiomycota	sp		0.4
Basid	Basidiomycota	sp	0.39	
Basid	Basidiomycota	sp	0.5	0.51
Basid	Cutaneotrichosporon	moniliiforme	-0.37	
Basid	Suillus	bovinus		0.38
Basid	Suillus	brunnescens	0.38	0.38
Basid	Suillus	variegatus		0.39
Chytr	Chytridiomycota	sp	0.4	
Chytr	Kochiomyces	sp	0.39	0.44
Chytr	Powellomyces	hirtus	0.39	0.38
Chytr	Rhizophydiales	sp		-0.4
Chytr	Rhizophydiales	sp	0.4	
Chytr	Spizellomycetales	sp	-0.37	
Chytr	Spizellomycetales	sp		-0.37
Glome	Funneliformis	caledonium		0.38
Glome	Funneliformis	mosseae	0.42	
Glome	Funneliformis	mosseae	0.38	
Glome	Glomeraceae	sp	0.37	
Glome	Glomeromycetes	sp	0.37	
Morti	Mortierella	globalpina	0.42	
Morti	Mortierella	sp	0.49	0.51
Morti	Mortierella	sp	-0.39	
Mucor	Umbelopsis	dimorpha		0.39
Neoca	Neocallimastigomycota	sp	0.38	0.38
Olpid	Olpidium	sp	0.38	
Rozel	Rozellomycota	sp	0.37	0.46
Rozel	Rozellomycota	sp		0.36
Rozel	Rozellomycota	sp	0.49	0.49
unide	Fungi	sp	0.39	0.46
unide	Fungi	sp	-0.39	
unide	Fungi	sp		0.37
unide	Fungi	sp	0.44	0.47

##### Microbial taxa abundance and soil OM% content

High organic matter content (OM%) of the soil correlated positively (*r* > 0.7, *P* < 0.001, Supplementary Table 5) with the abundance of Acidobacteriota (unidentified Acidobacteriae), Actinomycetota (unidentified Acidimicrobiia and Actinobacteria), Bacteroidota Bacteroidia, Chloroflexota KD4-96, Gemmatimonadota Gemmatimonadaceae, and Pseudomonadota (Alphaproteobacteria and Gammaproteobacteria) in bulk soil and rhizosphere. Low organic matter was associated with the abundance of Actinomycetota (Actinobacteria, thermoleophilia), Bacillota (Bacilli), Nitrospirota Nitrospiria, and Pseudomonadota (unidentified Alphaproteobacteria, Gammaproteobacteria), both in the bulk soil and rhizosphere (Supplementary Table 5).

The abundance of *Fusicolla* sp., *Mollisia* sp., *Mycosymbioces* sp., *Coniochaeta* sp., *Saitozyma* sp., unidentified *Powellomycetaceae*, and *Umbelopsis* sp. was positively associated with high OM%, whereas the abundance of *Metarhizium* sp., *Exophiala* sp., and unidentified *Mortierellaceae* was negatively associated with OM% in both rhizosphere and bulk soil (Supplementary Table 6).

## Discussion

### Beneficial and pathogenic microbes coexist in carrot rhizosphere and bulk soil

The results demonstrate that carrot rhizospheres and surrounding soils contain complex microbial communities comprising both beneficial and potentially pathogenic taxa. Samples were collected from 26 Finnish carrot fields, and neither high nor low storability was exclusively associated with the presence of pathogens or beneficial microbes. For instance, the major carrot pathogen *Mycocentrospora* sp. was associated with low storability, whereas other fungi commonly considered pathogenic (e.g. *Tilletiaria* sp. and *Plectosphaerella* sp.) were detected in fields containing carrots with both high and low storability. In this study, *Alternaria* spp. were associated with good carrot storability, and most *Alternaria* spp. are saprophytes commonly found in soil or on decaying plant tissues (Thomma 2003). However, *A. dauci, A. radicina* (which was neither found in our study nor previously detected in Finland; Latvala et al. 2024) and *A. alternata* are serious carrot pathogens worldwide. In addition, beneficial taxa such as *Trichoderma* spp., recognized for their biocontrol potential, were observed in all samples, irrespective of storability. These findings support the hypothesis that the rhizosphere and surrounding bulk soil harbour a dynamic microbial network in which beneficial and pathogenic organisms coexist (Abdelrazek et al. 2020, Kusstatscher et al. 2020). Abdelrazek et al. (2020) reported that carrot taproots host a diverse fungal endophytic community dominated by *Ascomycota* and largely consistent across management systems. However, organic farming increased endophytic richness relative to conventional systems. The reported taxa included pathogens such as *Alternaria dauci* and *Cercospora carotae*, alongside diverse endophytes, including *Ophiosphaerella, Ceratobasidium, Colletotrichum, Gibberella, Cladosporium, Aspergillus, Cyphellophora, Thanatephorus, Plectosphaerella, Rhizopycnis*, and *Phoma*, which together constitute key components of the carrot taproot fungal endophyte community (Abdelrazek et al. 2020). Here, the only fungal OTUs potentially assigned as endophytes and showing differential abundance between high- and low-storability carrot rhizospheres were *Mortierella* sp. and *Plectosphaerella* sp., classified along the pathotroph–saprotroph–symbiotroph continuum (DiLegge et al. 2019, Giraldo and Crous 2019).

### High microbial diversity might reduce the occurrence of postharvest diseases

In our study, postharvest disease incidence could not be predicted from the occurrence of any single microbial species. Although a high abundance of the pathogenic *Mycocentrospora* sp. was correlated with low storability, the overall patterns indicated that storability was not determined solely by the presence of carrot pathogens. This finding is consistent with previous research indicating that indigenous pathogens may become problematic only under conditions of reduced diversity or compositional shifts (Kusstatscher et al. 2020). The data presented here indicate that storability is multifactorial and influenced by microbial community structure rather than individual taxa. This finding aligns with the holobiont concept proposed for postharvest microbiomes (Wisniewski and Droby 2019).

Carrot diseases are predominantly fungal (Suojala 1999, Abdelrazek et al. 2020, Latvala et al. 2024). Here, the high fungal diversity in the rhizosphere was associated with a lower incidence of disease, thus suggesting a possible suppressive effect on postharvest pathogens. Similarly, an association was also observed between bacterial diversity and high storability, particularly when taxa such as *Streptomycetaceae, Bacillaceae*, and *Hyphomicrobiaceae* were present in high abundance. These groups are distinguished by their role in nutrient cycling, promotion of plant growth, and antagonism against pathogens through the production of antibiotics (Saxena et al. 2020, Alipour Kafi et al. 2021, Zhang et al. 2024).

Fungal antagonism extends beyond resource competition to include inducible chemical communication, such as volatile signalling triggered during direct confrontation, indicating an active chemical response rather than passive competition (Thomas et al. 2025). Indigenous soil and litter fungi from native forest ecosystems have been shown to mitigate abiotic stress and suppress soil-borne pathogens such as *Fusarium* in wheat (Ridout and Newcombe 2016), suggesting that natural soils represent an important reservoir of functionally relevant microbial taxa. These microbes may be introduced into agricultural systems via organic inputs and soil amendments or through dispersal from surrounding natural areas. Indeed, organic wood-derived amendments have been shown to enhance fungal activity in the rhizosphere, promote positive microbial interactions without favouring pathogens, and increase fungal biomass under field conditions (Clocchiatti et al. 2021), highlighting their potential to promote disease-suppressive soil communities. Moreover, high-quality cover crop residues with low C:N ratios can improve disease tolerance and microbiome function; major shifts in *Proteobacteria* and increased abundances of *Bacillaceae* and *Mortierellomycetes* have been reported (Liu et al. 2021). In our study, green manure use increased microbial diversity in soil but did not itself affect carrot storability. Although not statistically significant, the slightly lower storability of carrots from farms using green manure in the rotation may reflect multiple factors associated with organic farming rather than green manure alone; however, our data do not permit further analysis of this relationship.

However, diversity alone was not a universal predictor of carrot health, as some fields with diverse communities still exhibited disease. This suggests that other factors, such as soil physicochemical properties and crop management practices, are important for postharvest storability (Kinkel et al. 2012, Batista et al. 2024). As previously reported, rhizosphere microbial composition is shaped by interactions between biotic and abiotic factors that have substantial, context-dependent effects on rhizosphere microbiomes (Garbeva et al. 2008). In the boreal region, farming system and crop rotation have been associated with shifts in bacterial and fungal community composition, with organic cereal systems exhibiting greater autumn microbial richness, increased microbial activity and biomass, and more indicator taxa, including bacteria linked to soil and plant health, than conventional systems (Peltoniemi et al. 2021). Here, bacterial diversity was strongly driven by soil pH, a common pattern in acidic boreal agricultural soils (Fierer and Jackson 2006, Rousk et al. 2010), and was greater under organic farming. Fungal diversity was affected by weather conditions, such as rain and temperature, and by green manure use. This indicates that different farming practices may be required to support these diverse soil microbial communities.

### Good storability is associated with high abundance of beneficial microbial groups

The fields with high-storability carrots demonstrated consistent enrichment of beneficial taxa, including *Devosia* sp., which has been observed to associate with arbuscular mycorrhizal fungi (Zhang et al. 2024), and *Amycolatopsis* sp., which is renowned for producing antibacterial compounds (Kisil et al. 2021). Fungal genera such as *Metarhizium* and *Trichoderma* were linked to enhanced storability, presumably due to their well-known properties in promoting biocontrol and organic matter decomposition (Hermosa et al. 2012, Liao et al. 2014). Conversely, fields with low-storability carrots were dominated by microbes adapted to acidic, low-nutrient soils. While these microbes are not pathogenic, they lack strong disease-suppressive traits. For example, although some members of the Burkholderiaceae family (here associated with low storability) are pathogenic, the majority of them are known as plant growth-promoting soil bacteria, and some are even antagonistic to phytopathogenic bacteria and fungi (Coenye and Vandamme 2003). The results of this study suggest that soil remaining on the carrot surface is important for storability, and management practices aimed at fostering beneficial microbial groups and enhancing fungal diversity in soil could improve carrot storability (Larkin 2015, Kalam et al. 2020).

## Conclusion

This study demonstrates that carrot storability is shaped by the composition and diversity of soil and rhizosphere microbiomes rather than the presence of individual pathogens. High microbial diversity, particularly within fungal communities, combined with the predominance of functionally important bacterial taxa, is crucial for postharvest disease suppression and enhanced storability. It is recommended that future research endeavours focus on the exploration of functional interactions within these communities and on how to develop crop management practices to enhance postharvest quality.

## Supplementary Material

fiag102_Supplemental_Files

## References

[bib1] Abarenkov K, Zirk A, Piirmann T. et al. UNITE general FASTA release for Fungi, [Application/gzip]. [object Object]. 2021. 10.15156/BIO/1280049 (24 January 2022, date last accessed).

[bib2] Abdelrazek S, Choudhari S, Thimmapuram J. et al. Changes in the core endophytic mycobiome of carrot taproots in response to crop management and genotype. Sci Rep. 2020;10:13685. 10.1038/s41598-020-70683-x. PMC7426841 32792547

[bib3] Ali S, Tyagi A, Bae H. Plant microbiome: an ocean of possibilities for improving disease resistance in plants. Microorganisms. 2023;11:392. 10.3390/microorganisms11020392. PMC9961739 36838356

[bib4] Alipour Kafi S, Karimi E, Akhlaghi Motlagh M. et al. Isolation and identification of *Amycolatopsis* sp. Strain 1119 with potential to improve cucumber fruit yield and induce plant defense responses in commercial greenhouse. Plant Soil. 2021;468:125–45. 10.1007/s11104-021-05097-3.

[bib5] Anslan S, Bahram M, Hiiesalu I. et al. PipeCraft: flexible open-source toolkit for bioinformatics analysis of custom high-throughput amplicon sequencing data. Mol Ecol Resour. 2017;17:e234–40. 10.1111/1755-0998.12692. 28544559

[bib6] Batista BD, Wang J, Liu H et al. Biotic and abiotic responses to soilborne pathogens and environmental predictors of soil health. Soil Biol Biochem. 2024;189:109246. 10.1016/j.soilbio.2023.109246

[bib7] Bengtsson-Palme J, Ryberg M, Hartmann M. et al. Improved software detection and extraction of ITS1 and ITS 2 from ribosomal ITS sequences of fungi and other eukaryotes for analysis of environmental sequencing data. Methods Ecol Evol. 2013;4:914–9. 10.1111/2041-210X.12073.

[bib8] Berg G, Smalla K. Plant species and soil type cooperatively shape the structure and function of microbial communities in the rhizosphere: plant species, soil type and rhizosphere communities. FEMS Microbiol Ecol. 2009;68:1–13. 10.1111/j.1574-6941.2009.00654.x. 19243436

[bib9] Camacho C, Coulouris G, Avagyan V. et al. BLAST+: architecture and applications. BMC Bioinf. 2009;10:421. 10.1186/1471-2105-10-421. PMC2803857 20003500

[bib10] Caporaso JG, Lauber CL, Walters WA. et al. Ultra-high-throughput microbial community analysis on the Illumina HiSeq and MiSeq platforms. ISME J. 2012;6:8. 10.1038/ismej.2012.8. PMC3400413 22402401

[bib11] Chen L, Reeve J, Zhang L. et al. GMPR: a robust normalization method for zero-inflated count data with application to microbiome sequencing data. PeerJ. 2018;6:e4600. 10.7717/peerj.4600. PMC5885979 29629248

[bib12] Clocchiatti A, Hannula SE, Hundscheid MPJ. et al. Stimulated saprotrophic fungi in arable soil extend their activity to the rhizosphere and root microbiomes of crop seedlings. Environ Microbiol. 2021;23:6056–73. 10.1111/1462-2920.15563. PMC8596668 33973345

[bib13] Coenye T, Vandamme P. Diversity and significance of Burkholderia species occupying diverse ecological niches. Environ Microbiol. 2003;5:719–29. 10.1046/j.1462-2920.2003.00471.x. 12919407

[bib14] Das I . Fqgrep (Version 0.4.4) [Computer software]. 2011.

[bib15] DiLegge MJ, Manter DK, Vivanco JM. A novel approach to determine generalist nematophagous microbes reveals *Mortierella globalpina* as a new biocontrol agent against *Meloidogyne* spp. Nematodes Sci Rep. 2019;9:7521. 10.1038/s41598-019-44010-y. PMC6525257 31101887

[bib16] Directorate-General for Environment . Directive of the European parliament and of the council on Soil Monitoring and Resilience (Soil Monitoring Law). (Communication) COM(2023) 416 final, 2023. https://environment.ec.europa.eu/publications/proposal-directive-soil-monitoring-and-resilience_en (15 December 2025, date last accessed).

[bib17] Fierer N, Jackson RB. The diversity and biogeography of soil bacterial communities. Proc Natl Acad Sci. 2006;103:626–31. 10.1073/pnas.0507535103. PMC1334650 16407148

[bib18] Garbeva P, Van Elsas JD, Van Veen JA. Rhizosphere microbial community and its response to plant species and soil history. Plant Soil. 2008;302:19–32. 10.1007/s11104-007-9432-0.

[bib19] Giraldo A, Crous PW. Inside Plectosphaerellaceae. Stud Mycol. 2019;92:227–86. 10.1016/j.simyco.2018.10.005. PMC6276054 30518989

[bib20] Hermansen A, Wanner L, Nærstad R. et al. Detection and prediction of post harvest carrot diseases. Eur J Plant Pathol. 2012;133:211–28. 10.1007/s10658-011-9896-x.

[bib21] Hermosa R, Viterbo A, Chet I. et al. Plant-beneficial effects of Trichoderma and of its genes. Microbiology. 2012;158:17–25. 10.1099/mic.0.052274-0. 21998166

[bib22] Ihrmark K, Bödeker ITM, Cruz-Martinez K. et al. New primers to amplify the fungal ITS2 region—evaluation by 454-sequencing of artificial and natural communities. FEMS Microbiol Ecol. 2012;82:666–77. 10.1111/j.1574-6941.2012.01437.x. 22738186

[bib23] Kahala M, Blasco L, Joutsjoki V. Molecular characterization of spoilage bacteria as a means to observe the microbiological quality of carrot. J Food Prot. 2012;75:523–32. 10.4315/0362-028X.JFP-11-185. 22410227

[bib24] Kalam S, Basu A, Ahmad I et al. Recent Understanding of Soil Acidobacteria and Their Ecological Significance: A Critical Review. Front Microbiol. 2020;11:580024. 10.3389/fmicb.2020.580024 PMC7661733 33193209

[bib25] Karisto P, Latvala S, Haapalainen M. et al. Carrot storage diseases predominantly caused by mixed fungal infections. Eur J Plant Pathol. 2026. 174;1829–42. 10.1007/s10658-026-03240-3

[bib26] Kassambara A, Mundt F. factoextra: extract and visualize the results of multivariate data analyses. 2020. http://www.sthda.com/english/rpkgs/factoextra.

[bib27] Kinkel LL, Schlatter DC, Bakker MG. et al. Streptomyces competition and co-evolution in relation to plant disease suppression. Res Microbiol. 2012;163:490–9. 10.1016/j.resmic.2012.07.005. 22922402

[bib28] Kisil OV, Efimenko TA, Efremenkova OV. Looking back to *Amycolatopsis*: history of the antibiotic discovery and future prospects. Antibiotics. 2021;10:1254. 10.3390/antibiotics10101254. PMC8532670 34680834

[bib29] Kõljalg U, Nilsson RH, Abarenkov K. et al. Towards a unified paradigm for sequence-based identification of fungi. Mol Ecol. 2013;22:5271–7. 10.1111/mec.12481. 24112409

[bib30] Kusstatscher P, Cernava T, Abdelfattah A. et al. Microbiome approaches provide the key to biologically control postharvest pathogens and storability of fruits and vegetables. FEMS Microbiol Ecol. 2020;96:fiaa119. 10.1093/femsec/fiaa119. 32542314

[bib31] Kusstatscher P, Cernava T, Harms K. et al. Disease incidence in sugar beet fields is correlated with microbial diversity and distinct biological markers. Phytobiomes J. 2019;3:22–30. 10.1094/PBIOMES-01-19-0008-R.

[bib32] Lahti L, Shetty S. Microbiome R package [Computer software]. Bioconductor. 2019. 10.18129/B9.BIOC.MICROBIOME.

[bib33] Lal R. Restoring soil quality to mitigate soil degradation. Sustainability. 2015;7:5875–95. 10.3390/su7055875.

[bib34] Larkin RP. Soil health paradigms and implications for disease management. Annu Rev Phytopathol. 2015;53:199–221. 10.1146/annurev-phyto-080614-120357. 26002292

[bib35] Latvala S, Haapalainen M, Karisto P. et al. Changes in the prevalence of fungal species causing post-harvest diseases of carrot in Finland. Ann Appl Biol. 2024;185:23–35. 10.1111/aab.12908.

[bib36] Lê S, Josse J, Husson F. FactoMineR : an R Package for Multivariate Analysis. J Stat Softw. 2008;25. 10.18637/jss.v025.i01.

[bib37] Liao X, O’Brien TR, Fang W. et al. The plant beneficial effects of Metarhizium species correlate with their association with roots. Appl Microbiol Biotechnol. 2014;98:7089–96. 10.1007/s00253-014-5788-2. 24805846

[bib38] Liu X, Hannula SE, Li X. et al. Decomposing cover crops modify root-associated microbiome composition and disease tolerance of cash crop seedlings. Soil Biol Biochem. 2021;160:108343. 10.1016/j.soilbio.2021.108343.

[bib39] Love MI, Huber W, Anders S. Moderated estimation of fold change and dispersion for RNA-seq data with DESeq2. Genome Biol. 2014;15:550. 10.1186/s13059-014-0550-8. PMC4302049 25516281

[bib40] Martin M. Cutadapt removes adapter sequences from high-throughput sequencing reads. EMBnetJournal. 2011;17:10. 10.14806/ej.17.1.200.

[bib41] McMurdie PJ, Holmes S. phyloseq: an R package for reproducible interactive analysis and graphics of microbiome census data. PLoS One. 2013;8:e61217. 10.1371/journal.pone.0061217. PMC3632530 23630581

[bib42] Mendes R, Garbeva P, Raaijmakers JM. The rhizosphere microbiome: significance of plant beneficial, plant pathogenic, and human pathogenic microorganisms. FEMS Microbiol Rev. 2013;37:634–63. 10.1111/1574-6976.12028. 23790204

[bib43] Mendes R, Kruijt M, De Bruijn I. et al. Deciphering the rhizosphere microbiome for disease-suppressive bacteria. Science. 2011;332:1097–100. 10.1126/science.1203980. 21551032

[bib44] Mesny F, Hacquard S, Thomma BP. Co-evolution within the plant holobiont drives host performance. EMBO Rep. 2023;24:e57455. 10.15252/embr.202357455. PMC10481671 37471099

[bib45] Nguyen NH, Song Z, Bates ST. et al. FUNGuild: an open annotation tool for parsing fungal community datasets by ecological guild. Fungal Ecol. 2016;20:241–8. 10.1016/j.funeco.2015.06.006.

[bib46] O’Donnell K, Whitaker BK, Laraba I. et al. DNA sequence-based identification of *Fusarium*: a work in progress. Plant Dis. 2022;106:1597–609. 10.1094/PDIS-09-21-2035-SR. 34907805

[bib47] Oksanen J, Blanchet FG, Friendly M et al. vegan: Community Ecology Package 2020, (Version R package version 2.5-7.) [Computer software]. 2020. https://CRAN.R-project.org/package=vegan

[bib48] Oren A, Arahal DR, Rosselló-Móra R. et al. Emendation of Rules 5b, 8, 15 and 22 of the International Code of Nomenclature of Prokaryotes to include the rank of phylum. Int J Syst Evol Microbiol. 2021;71:6. 10.1099/ijsem.0.004851 34161220

[bib49] Oren A, Mareš J, Rippka† R. Validation of the names Cyanobacterium and Cyanobacterium stanieri, and proposal of Cyanobacteriota phyl. Nov. Int J Syst Evol Microbiol. 2022;72:10. 10.1099/ijsem.0.005528 36251754

[bib50] Pankhurst, Doube, & Gupta . Pankhurst CE, Doube BM, Gupta VVSR, (eds.) Biological Indicators of Soil Health. Wallingford, UK: CAB International, 1997.

[bib51] Peltoniemi K, Velmala S, Fritze H. et al. Long-term impacts of organic and conventional farming on the soil microbiome in boreal arable soil. Eur J Soil Biol. 2021;104:103314. 10.1016/j.ejsobi.2021.103314.

[bib52] Pereira P, Bogunovic I, Muñoz-Rojas M. et al. Soil ecosystem services, sustainability, valuation and management. Curr Opin Environ Sci Health. 2018;5:7–13. 10.1016/j.coesh.2017.12.003.

[bib53] Pinheiro JC, Bates DM, DebRoy S et al. nlme: Linear and Nonlinear Mixed Effects Models (Version 3.1-164) [Computer software]. 2023. https://CRAN.R-project.org/package=nlme

[bib54] Quast C, Pruesse E, Yilmaz P. et al. The SILVA ribosomal RNA gene database project: improved data processing and web-based tools. Nucleic Acids Res. 2012;41:D590–6. 10.1093/nar/gks1219. PMC3531112 23193283

[bib55] Que F, Hou X-L, Wang G-L. et al. Advances in research on the carrot, an important root vegetable in the Apiaceae family. Hortic Res. 2019;6:69. 10.1038/s41438-019-0150-6. PMC6544626 31231527

[bib56] Raaijmakers JM, Paulitz TC, Steinberg C. et al. The rhizosphere: a playground and battlefield for soilborne pathogens and beneficial microorganisms. Plant Soil. 2009;321:341–61. 10.1007/s11104-008-9568-6.

[bib57] R Core Team . R: A language and environment for statistical computing, [Computer software], 2024. https://www.R-project.org/.

[bib58] Ridout M, Newcombe G. Disease suppression in winter wheat from novel symbiosis with forest fungi. Fungal Ecol. 2016;20:40–8. 10.1016/j.funeco.2015.10.005.

[bib59] Rognes T, Flouri T, Nichols B. et al. VSEARCH: a versatile open source tool for metagenomics. PeerJ. 2016;4:e2584. 10.7717/peerj.2584. PMC5075697 27781170

[bib60] Rousk J, Bååth E, Brookes PC. et al. Soil bacterial and fungal communities across a pH gradient in an arable soil. ISME J. 2010;4:1340–51. 10.1038/ismej.2010.58. 20445636

[bib61] Saxena AK, Kumar M, Chakdar H. et al. Bacillus species in soil as a natural resource for plant health and nutrition. J Appl Microbiol. 2020;128:1583–94. 10.1111/jam.14506. 31705597

[bib62] Schloss PD, Westcott SL, Ryabin T. et al. Introducing mothur: open-Source, platform-independent, community-supported software for describing and comparing microbial communities. Appl Environ Microbiol. 2009;75:7537–41. 10.1128/AEM.01541-09. PMC2786419 19801464

[bib63] Suojala T. Effect of harvest time on the storage performance of carrot. J Hortic Sci Biotechnol. 1999;74:484–92. 10.1080/14620316.1999.11511141.

[bib64] Thomas GA, Vuts J, Withall DM. et al. Inducible Volatile chemical signalling drives antifungal activity of *Trichoderma hamatum* GD12 during confrontation with the pathogen *Sclerotinia sclerotiorum*. Environ Microbiol Rep. 2025;17:e70192. 10.1111/1758-2229.70192. PMC12462542 40996658

[bib65] Thomma BPHJ. Alternaria spp.: from general saprophyte to specific parasite. Mol Plant Pathol. 2003;4:225–36. 10.1046/j.1364-3703.2003.00173.x. 20569383

[bib66] Vuorinen J, Mäkitie O. Department of Soil Science, Agricultural Research Centre of Finland, The Method of Soil Testing in Use in Finland. Helsinki: Agrogeological Publications, 1955;63:1–44.

[bib67] Wei T, Simko V. R package ‘corrplot’: visualization of a Correlation Matrix. (Version Version 0.95.) [Computer software]. 2024. https://github.com/taiyun/corrplot

[bib68] White TJ, Bruns T, Lee S et al. 38—amplification and direct sequencing of fungal ribosomal rna genes for phylogenetics. In: Innis MA, Gelfand DH, Sninsky JJ, and White TJ (eds.), PCR Protocols. San Diego, CA: Academic Press, 1990; 315–22. 10.1016/B978-0-12-372180-8.50042-1

[bib69] Wickham H . ggplot2: Elegant Graphics for Data Analysis. Cham, Switzerland: Springer International Publishing. 2016. 10.1007/978-3-319-24277-4

[bib70] Wisniewski M, Droby S. The postharvest microbiome: the other half of sustainability. Biol Control. 2019;137:104025. 10.1016/j.biocontrol.2019.104025.

[bib71] Zhang C, Van Der Heijden MGA, Dodds BK. et al. A tripartite bacterial-fungal-plant symbiosis in the mycorrhiza-shaped microbiome drives plant growth and mycorrhization. Microbiome. 2024;12:13. 10.1186/s40168-023-01726-4. PMC10799531 38243337

